# Perception-based constraint solving for sudoku images

**DOI:** 10.1007/s10601-024-09372-9

**Published:** 2024-10-05

**Authors:** Maxime Mulamba, Jayanta Mandi, Ali İrfan Mahmutoğulları, Tias Guns

**Affiliations:** 1 Data Analytics Laboratory, Vrije Universiteit Brussel, Pleinlaan 5, Brussels, 1050 Belgium; 2 KU Leuven, Department of Computer Science, Celestijnenlaan 200A, Leuven, 3001 Belgium

**Keywords:** Constraint programming, Machine learning, Visual sudoku, Joint inference

## Abstract

We consider the problem of *perception-based constraint solving*, where part of the problem specification is provided *indirectly* through an image provided by a user. As a pedagogical example, we use the complete image of a Sudoku grid. While the rules of the puzzle are assumed to be known, the image must be interpreted by a neural network to extract the values in the grid. In this paper, we investigate (1) *a hybrid modeling approach* combining machine learning and constraint solving for *joint inference*, knowing that blank cells need to be both predicted as being blank and filled-in to obtain a full solution; (2) the effect of *classifier calibration* on joint inference; and (3) how to deal with cases where the constraints of the reasoning system are not satisfied. More specifically, in the case of handwritten *user errors* in the image, a naive approach fails to obtain a feasible solution even if the interpretation is correct. Our framework *identifies* human mistakes by using a constraint solver and helps the user to *correct* these mistakes. We evaluate the performance of the proposed techniques on images taken through the Sudoku Assistant Android app, among other datasets. Our experiments show that (1) joint inference can correct classifier mistakes, (2) overall calibration improves the solution quality on all datasets, and (3) estimating and discriminating between user-written and original visual input while reasoning makes for a more robust system, even in the presence of user errors.

## Introduction

Symbolic and sub-symbolic approaches are the two primary branches of artificial intelligence (AI). Symbolic AI aims to embed explicit knowledge into AI agents through symbolic description [1], such as propositional or first-order logic. Sub-symbolic AI, as opposed to symbolic AI, represents semantic entities through numerical vector representations, rather than human-understandable concepts [2]. Sub-symbolic AI strives to build intelligent agents by learning associations from observations through statistical learning, with minimal or no prior knowledge, typically relying on large-scale data [3]. In the last decade, the success of deep learning in the presence of big data has made sub-symbolic AI the dominant branch of AI. Deep learning has achieved excellent results in various tasks, such as object detection [4], speech recognition [5], image recognition [6] and more [7].

State-of-the-art deep learning techniques, such as Convolutional Neural Networks (CNN), can perform a variety of perceptual tasks that involve finding patterns in sensory data as well as or better than people. However, perceptual accuracy alone may not lead to the desired application-specific results in practice [8]. This is especially true for problems that require applying symbolic and logical reasoning to real-life situations. To illustrate, consider the example of scene graph parsing [9], where an AI agent needs to identify semantic relationships between entities in a given image. Although specialized deep CNNs are able to recognize and interpret the entities, they fail to rule out predictions that violate common sense reasoning, as can be achieved through a symbolic approach.

To tackle such problems, recent years have seen growing interest in neuro-symbolic methods, which **integrate neural perception with symbolic reasoning**. These hybrid methods allow for explicit modeling of prior knowledge, which can come from years of accumulated domain knowledge and common sense knowledge. Thus, they have several advantages. For example, incorporating a reasoning module on top of neural network predictions alleviates the burden of learning this prior knowledge from data. As a result, integrated neuro-symbolic approaches can perform symbolic reasoning over semantic entities, whereas a pure ML approach can fail to learn to do so, even with a large amount of training data [10]. Another important aspect of neuro-symbolic reasoning is that the explicit modeling of knowledge provides interpretability, leading towards explainable AI [11].

**Fig. 1:**
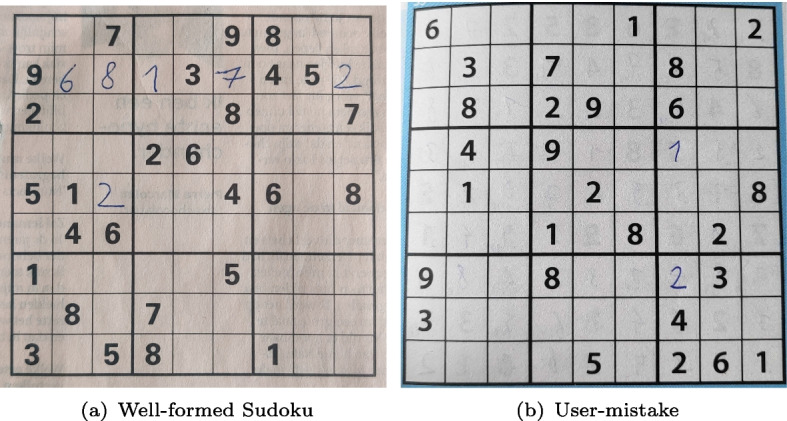
Images of Sudoku puzzles taken by a smartphone camera using the Sudoku assistant Android app

As a pedagogical example of integrating learning with reasoning, we consider the task of solving *visual Sudokus*, i.e., solving the Sudoku puzzles from their images Fig. 1. For this purpose, digits in the images must be identified first. Here, identifying digits in each cell forms the *perception task* and coming up with a solution that adheres to all Sudoku constraints, is the *reasoning tas*k. We remark that we do *not* want to learn the constraints of Sudokus from images [12–14]; rather, we consider that constraints are known beforehand and from given images we want to generate Sudoku solutions, which satisfy the constraints. We also assume a fully supervised setting, in which fully labeled instances are given as training data to learn from. Our approach can be adapted to many real-world settings, where humans can encode their logical interpretation as constraints and the goal is to develop an AI agent augmenting neural perception with human intelligence. Examples include common sense reasoning in autonomous driving systems [15], or complex motion planning from visual input in robotics [16].

While Large Language Models (LLM) are becoming more consistent at solving reasoning problems, they suffer from hallucination and can produce solutions that do not adhere to the rules of Sudoku. We provide a discussion on LLMs as a candidate approach for Visual Sudoku Solving in the appendix.

**Fig. 2:**
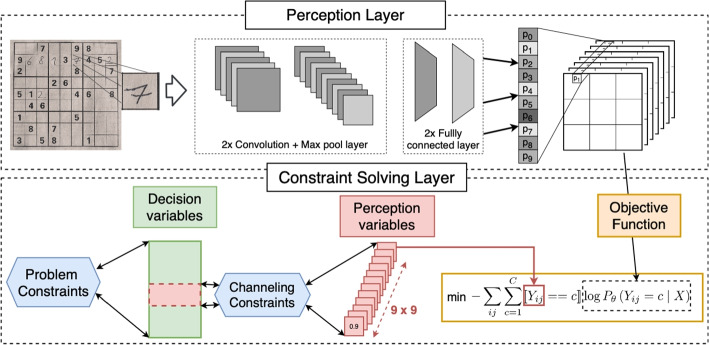
Overview of our perception-based constraint solving approach

The most straightforward approach to tackle this visual Sudoku solving problem is to treat the perception and reasoning tasks separately. In such an approach, the digit of each cell is predicted first and then the Sudoku is solved using the predictions. In our conference paper [17], we show that a *joint inference approach*, which integrates symbolic reasoning with probabilistic predictions during inference, performs significantly better. The motivation behind the *joint inference approach* is to identify mistakes made by the network in the perception task, by detecting violations of the symbolic constraints and correcting the mistakes while adhering to the symbolic constraints. We also demonstrate how this solving approach can be strengthened by reasoning over *higher-order constraints* of the puzzle. We present an overview of the joint inference approach in Fig. 2.

However, in that conference paper, we adopted an experimental setup from the work by [13]. In that setup, all the numbered cells are filled with MNIST [18] images of digits, and the blank cells are known. Note that digit recognition in the MNIST dataset is a relatively easy perception task as many deep learning architectures can make predictions with accuracy higher than $99\%$. We observed that even with such accuracy, a naive integration of perception and reasoning would fail to solve the task for some instances. Additionally, as the digit of each cell is provided as a distinct image, the perception task does not need to deal with the challenge of identifying the location of each cell. Finally, many MNIST-based setups for visual sudoku assume that positions of blank cells are provided beforehand.

In this work, we go further by dealing with real-world images of Sudoku grids, taken with a smartphone through the Sudoku Assistant Android app [19]. For instance, consider the images shown in Fig. 1(a). Since the entire image of a Sudoku is considered as input, the perception task consists of identifying all 81 cells present in the image, detecting empty cells, and predicting digits in the non-empty cells.

Furthermore, we also investigate images of *pen-and-paper* Sudokus that people started filling in by hand. As Fig. 1(b) shows with the hand-written ‘2’ in the lower right, this means that a human might make mistakes while trying to solve the Sudoku. These mistakes are conceptually different from a prediction mistake made by the neural network, as an error that a human makes will lead to an unsolvable problem even if the perception is 100% correct.

Handling such errors is essential for an AI Assistant that interacts with users and aim to provide meaningful feedback. We propose different ways to handle this, both at the perception and reasoning levels.

Our work introduces a modular framework for perception-based constraint solving, that operates independently of CNN training strategies. This modularity enables the use of our work along with various CNN architectures, namely those focused on single-cell processing or full-image analysis, as well as with other neuro-symbolic reasoning frameworks. We evaluate these different approaches and use cases in the experiments.

***Contributions***

We propose the following contributions to tackle the problem of solving constraint problems with perception-based input:

- Working with real-world images for perception-based constraint solving tasks presents various challenges, including selecting a suitable architecture and coping with class imbalance. We propose several ways to pre-train the neural networks, effectively handling such challenges for the Sudoku use case.
- We experimentally evaluate our approaches using three Sudoku datasets, demonstrating that the baseline approach outputs infeasible solutions in more than 20% of Sudoku images, which are corrected by the joint inference approach. Additionally, we compare our approach with state-of-the-art methods, illustrating how they can be enhanced by joint inference.
- Furthermore, we consider pen-and-paper Sudokus, where human users attempt to solve printed Sudoku puzzles. Recognizing the possibility of mistakes made by users, we introduce and demonstrate effective approaches to obtain the correct Sudoku solution, even in the presence of errors made by users.

The rest of the paper is structured as follows. After providing an overview of the related works in Section 2, in Section 3, we provide a background on constraint satisfaction problems (CSPs) to establish foundational knowledge before formulating Sudoku as a CSP. Then we introduce the motivation behind using a neural network classifier to solve a visual Sudoku problem. Next, in Section 4, we explore neural network architecture design choices for recognizing digits and the use of calibration techniques. In Section 5, we formulate the notion of joint inference for perception-based constraint solving and propose a generic approach to integrate probabilistic predictions into the constraint reasoning task. In Section 6, we investigate how to improve predictions in the presence of inputs by human users and correct their mistakes. We then experimentally validate the proposed contributions on images of Sudoku puzzles in Section 7. Finally, we conclude with some ideas for future work.

***Publication history***

This article is an extension of a previous conference paper [17]. The current paper extends the previous paper with a *generic* approach considering the whole image of a Sudoku as an input, as well as an approach for **handling user mistakes** that violate the assumptions of the constraints. This article considers a wider range of CNN architectures, and provides an empirical evaluation of our framework on three datasets, as opposed to one previously.

## Related works

There has been a growing interest in incorporating prior knowledge or problem-specific constraints into the training of neural networks. Recent surveys [20–22] provide an extensive overview of the work done in this body of research.

***Neuro-symbolic AI for perception-based reasoning***

Several methods aim to combine deep neural networks and symbolic solvers to address perception-based reasoning tasks such as visual jigsaw puzzles [23, 24], image-based planning problems [25], or visual question answering [26, 27]. Neuro-symbolic approaches have also successfully addressed more complex tasks that require object-centric perception such as NSFR [28], or Faster-LTN [29]. Our work is closely related to neuro-symbolic systems built to tackle hand-written equation or sentence recognition, by considering a reasoning that enforces hard constraints over the output of a perception layer, such as DeepProbLog [30], Nester [31], (G)ABL [32] or the Apperception engine [33]. These often assume a semi-supervised setting, where no (or little) direct supervision on the perception is provided. Also often relatively simple combinatorial problems/constraints are used. We do assume a supervised setting, and consider larger finite-domain combinatorial problems. Contrary to ours, none of the above-mentioned works explicitly addresses the challenge of human mistakes in the input.

***Visual Sudoku***

In recent years, the combination of deep learning for image perception and symbolic reasoning to solve visual Sudoku has become increasingly popular. Some systems jointly learn the constraints and to classify cell values [13, 14, 34, 35]. However, in our work, we assume a fully-supervised setting, where constraints are explicitly declared through the solver. Another common limitation, also present in [17, 36], is that instances are built by sampling cell images from MNIST [37], on which recent machine learning approaches achieve near-perfect accuracy [38]. The problem can easily be made more difficult by sampling cells from more challenging image datasets [39]. Images from a phone camera are in RGB space, can contain artifacts such as grid borders and are overall noisier depending on camera angle or lighting conditions. Therefore, both image perception and symbolic reasoning with these images present a significant challenge. The work in [40] extends this setting further by considering uncropped RGB images of Sudoku. This requires addressing the additional task of locating the puzzle on the image, either with established image processing algorithms or through a dedicated neural network, which is out of the scope of this work.

Many related approaches, including our conference article [17], work with synthetic Visual Sudokus built by sampling MNIST [41] digits for each cell. Thus they assume that the locations of non-empty cells (hereafter referred to as ‘givens’) are known. This creates an easier setup, where the framework does not have to handle empty cells, both in prediction and reasoning. In [34], a variant of the MNIST-based setting is considered where instances are extracted from an online Sudoku generator. As such, they relax the usual assumption that given locations are known. Instead, the hybrid system receives a full image as input and needs to identify blank and filled cells – which constitute the initial puzzle – before finding a solution. This setup is more representative of real-life use cases, wherein the system does not have access to given location information [19].

The work most closely related to ours is NASR [42], a hybrid system that addresses image-based reasoning tasks. NASR achieves this by combining machine learning and constraint solving through four primary components: a perception module that provides probability distributions for each cell in the input image, a neural solver that learns to predict a solution from probability distributions, a mask predictor that learns to eliminate cells in the predicted output violating Sudoku constraints, and a symbolic solver that reasons over the masked predicted output to find a feasible solution. The incorporation of a mask predictor comes from the need for the neural solver to produce feasible solutions during end-to-end training. Instead, we alleviate the burden of training multiple constraint-aware neural networks by considering a post-hoc reasoning scheme that jointly finds a solution and corrects erroneous puzzle interpretation made by the perception module.

## Background

One advantage of symbolic reasoning is the ability to reason about relationships between different variables. In many real-world applications of AI such as scheduling and configuration problems, the AI agent is expected to deliver outputs satisfying some known constraints. Constraint programming (CP) is a highly powerful paradigm for modeling and solving problems that involve constraints [43]. Such problems can be represented in CP as constraint satisfaction problems (CSPs). In this section, we first model the symbolic reasoning task as a CSP, i.e., reasoning about the rules of the Sudoku puzzle. Next, we formalize the visual perception task as a machine learning problem.

### Constraint satisfaction problems

A *CSP* is formulated as a triplet $(\mathcal {V},\mathcal {D},\mathcal {C})$, where $\mathcal {V}$ is a set of decision variables, each of which has its possible values in a domain contained in the set $\mathcal {D}$, and $\mathcal {C}$ is a set of constraints that need to be satisfied over the variables in $\mathcal {V}$. In most cases, we are not only interested in knowing whether a constrained problem is solvable, but we want the *best* possible solution according to an objective.

A *constraint optimisation problem* (*COP*), formulated as $COP(\mathcal {V},\mathcal {D},\mathcal {C},\mathcal {L})$, finds a feasible solution *S* of optimum value $\mathcal {L}(S)$ with respect to a function $\mathcal {L}$ over the variables. In case of a *minimisation* problem, we have $S \in COP(\mathcal {V},\mathcal {D},\mathcal {C},\mathcal {L})$ iff $S \in CSP(\mathcal {V},\mathcal {D},\mathcal {C})$ and $\not \exists ~T \in CSP(\mathcal {V},\mathcal {D},\mathcal {C})$ with $\mathcal {L}(T) < \mathcal {L}(S)$. In this setting, we commonly refer to $\mathcal {L}$ as a *cost* function.

***Example: Sudoku***

In this work, we consider a prototype CSP, namely the $9\times 9$ Sudoku. Sudoku is a number puzzle, played on a partially filled $9 \times 9$ grid. The goal is to figure out the unique solution by filling in the blank cells with numbers from 1 to 9 in such a way that each row, each column, and each of the nine $3 \times 3$ subgrids contain all the numbers from 1 to 9 once and only once.

The Sudoku can be represented as a $CSP(\mathcal {V},\mathcal {D},\mathcal {C})$ where:

- $\mathcal {V}$ is the set of 81(=$9 \times 9$) variables $\mathcal {V}_{\texttt{rc}}$ $(\texttt{r,c} \in \{1,...,9\})$ for every cell at row *r* and column *c* in the grid, and
- For each $\mathcal {V}_{\texttt{rc}} \in \mathcal {V}$, its domain $\mathcal {D}(\mathcal {V}_{\texttt{rc}}) = \{1,...,9\}$.
- The solution to a given Sudoku must satisfy two sets of constraints. The first set of constraints, $\mathcal {C}_{rules}$, defines the rules of Sudokus. $\mathcal {C}_{rules}$ consists of the following constraints:
  1 $$\begin{aligned} \begin{aligned} \forall&\  &r\in &   \{1, \ldots , 9\}&\ \mathtt { alldifferent(\mathcal {V}_{\texttt{r1}}, \ldots ,\mathcal {V}_{\texttt{r9}})} \\ \forall  &   c\in &   \{1, \ldots , 9\}&\ \mathtt {alldifferent( \mathcal {V}_{\texttt{1c}}, \ldots , \mathcal {V}_{\texttt{9c}})}\\ \forall  &   r, c&\!\in \!&\{1,4,7\}&\ \mathtt {alldifferent(\mathcal {V}_{r c}, \ldots , \mathcal {V}_{(r\!+\!2) c}, \mathcal {V}_{r(c\!+\!1)}, \ldots , \mathcal {V}_{(r+2)(c\!+\!1)},}\\  &      &   &\mathtt {\mathcal {V}_{r(c+2)}, \ldots , \mathcal {V}_{(r+2)(c+2)})} \end{aligned} \end{aligned}$$
  The second set of constraints, $\mathcal {C}_{given}$, assigns the given values to each of the non-empty cells, referred to as givens. If $\{ \mathcal {V}_{rc} \}^{given} \subset \mathcal {V}$ is the set of givens, with $\{ \mathtt {y_{rc}} \}^{given}$ being the set of given values, $\mathcal {C}_{given}$ consists of a set of assignments, i.e.,
  $$ \mathcal {V}_{rc} = \mathtt {y_{rc}}, \quad \forall \ \mathcal {V}_{rc} \in \{ \mathcal {V}_{rc} \}^{given} \subset \mathcal {V}$$

Since the set of variables $\mathcal {V}$ and the domain $\mathcal {D}$ remain same for all Sudokus, for notational convenience, we denote a Sudoku by $CSP(\mathcal {C}_{rules} \wedge \mathcal {C}_{given})$.

### Machine learning for perception-based constraint solving

The focus of our work is to solve a decision problem from raw sensor input, such as images. In this setting, solving the problem consists of a perception task followed by a reasoning task. The former corresponds to identifying target objects within a region-of-interest in the image. The reasoning task, framed as a CSP, is handled by a dedicated solver. For the perception task, we use a (probabilistic) neural network classifier. In the following subsection, we briefly describe how a generic probabilistic classifier is trained.

***Probabilistic classifier***

The goal of a classifier is to correctly assign a given instance to a class label. In a supervised machine learning problem, the classifier is trained using a training dataset. The training dataset is denoted by $\mathcal {I}_{train} = \{(X_i,y_i)\}_{i=1}^n$ with $X_i \in \mathbb {R}^d$ and $y_i \in \mathbb {R}$ being the feature vector and class label respectively. The goal of a classifier is to learn a function approximator that predicts the label of an unseen instance from its feature vector *X*, denoted as $f_\theta (X)$, where $\theta$ represents the trainable parameters of the learning function. In the case of a probabilistic classifier, the output is the predicted probability of *y* belonging to class *k*, i.e., $P_\theta (y = k \ | X)$. After predicting the class probabilities, the class with the highest probability may be assigned as the class label, i.e., $\hat{y} = f_\theta (X) = \mathop {\mathrm {arg\,max}}\limits _{k} P_\theta (y = k | X)$ [44].

The model is trained to learn parameters $\theta$ so that it can generalize from the training data to make accurate classifications on new, unseen instances. To quantify the accuracy of classification, a loss function is defined, which measures the deviation between the model prediction and the true class label. For a given (*X*, *y*) pair, the loss function can be expressed as $\mathcal {J}(f_\theta (X),y)$. An example of a loss function for probabilistic classifiers with $K$ possible classes is the *cross-entropy loss*, shown below:

2 $$\begin{aligned} \mathcal {J}_{\text {CE}}(f_\theta (X), y) = - \sum _{k=1}^K\llbracket y = k \rrbracket \log P_\theta (y = k | X) \end{aligned}$$

The indicator function defined by Iverson brackets $\llbracket .\rrbracket$ is equal to 1 for values of *y* and *k* for which the statement is true, and 0 otherwise. Therefore, minimizing the cross-entropy loss translates to maximizing the probability of assigning the instance to its correct class label. In supervised learning, the parameter $\theta$ is estimated by minimizing the average loss on the training dataset, augmented by a regularizer function $\Omega$, which prevents overfitting. Formally, the training loss can be expressed as below:

3 $$\begin{aligned} \dfrac{1}{n} \sum _{i=1}^n \mathcal {J}_{\text {CE}}(f_\theta (X_i), y_i) + \Omega (\theta ) \end{aligned}$$

***Example: visual Sudoku***

In the visual Sudoku problem, the purpose of using a neural network classifier is to identify all the givens of the Sudoku grid and recognize all the digits in the givens. In this setting, a classifier $f_\theta$ takes a full-image *X* as a feature vector, and returns $\hat{y} \in \mathbb {R}^{9\times 9}$. Each of the 81 predictions in $\hat{y}$ is either *blank* or a number between 1 and 9. A solution to the Sudoku can be found by solving a perception-based CSP, namely $\texttt {PBCSP}(\mathcal {C}_{rules}, X, \theta )$ . Note that in this expression, $\mathcal {C}_{given}$ is not explicitly included, because in visual Sudokus, the locations of the givens are not known beforehand. It is possible to infer the givens from the predicted label $\hat{y}$, and to generate the predicted label $\hat{y}$, we may use a trained probabilistic classifier. A trained probabilistic classifier provides a probability vector for each of the 81 cells. From this $\hat{y}$ could be generated by taking the argmax value of the probability vector in each cell. Then we could define $\mathcal {C}_{given}$ by assigning the decision variables in the cells classified as givens to the predicted numbers. Thereafter we could find a solution filling the blank cells by solving $CSP(\mathcal {C}_{rules} \wedge \mathcal {C}_{given})$. But we should bear in mind that the predictions may be inaccurate and this approach, we have referred to as the *baseline* in [17], suffers from a shallow integration of machine learning output into the constraint reasoning.

The weakness of this approach is that even if there is a near-perfect classifier, $f_\theta$, which can predict the value of a single digit with very high accuracy, it still has to predict 81 digits. Consequently, the probability of making at least one error is not negligible. As an example, consider a machine learning classifier with $99\%$ accuracy on digit recognition. Using the chain rule, the joint probability of correctly predicting values for the whole grid is $0.99^{81} \approx 44.3\%$. This is problematic because even one misclassified cell can lead to an unsolvable problem since the classifier does not take into account the constraints of the problem when generating predictions. The problem remains even if we increase predicting accuracy. Following the same logic, a $99.9\%$-accurate classifier would correctly predict around $92\%$ instances.

In Section 5, we investigate how to better integrate the probabilistic output of a neural network classifier into the reasoning allowing to correct errors, including the errors in identifying the blank cells, made by the classifier. Furthermore, we also consider Sudoku images, which are partially filled by a human. Note that in such scenarios, not only a classifier but also a human can make mistakes.

The system should be able to identify and correct mistakes that were introduced by a user.

In Section 6, we explore how to handle mistakes that were introduced by a human for prediction and solving.

## Key considerations in training neural networks for perception-based constraint solving

The first stage of the perception-based constraint solving task is to develop the perception module. As alluded before, nowadays, the perception module typically consists of a neural network. The design of the neural network is tailored specifically to the perception task at hand. In this section, we explain the design choices made for the neural network in tackling the Visual Sudoku problem.

### DNN architecture

A crucial aspect of handling real-world Sudoku images is the classification of all the $9 \times 9$ cells given a roughly cropped image of the grid. Since we assume we can extract a cropped image of the sudoku grid, from a picture of a newspaper page [40], the purpose of the perception layer is to have a network that produces $9 \times 9$ probabilistic outputs over the class labels for each cell. The subsequent sub-sections introduce possible choices of architecture for neural networks in our context.

#### Perception level

Related approaches for perception based constraint solving consider several CNN architectures as perception modules [14, 34, 39, 42]. Their first characteristic is the level at which they perform inference. Namely, *Whole-CNN* process the entire input image at once to output a discrete probability distribution for each cell, whereas *Cell-CNN* process patches of the input images.

***Whole-image classification with convolutional neural networks***

As an example, the NeurASP framework [34] considers a single Convolutional neural network (CNN) that takes an image as input, outputs a matrix $\in \mathbb {R}^{81\times 10}$, representing the probability distribution of possible values for each cell. This network has 5 convolution blocks (each block consists of a convolutional layer, batch normalisation, dropout and ReLU activation) followed by max-pooling, a 1x1 convolution and a softmax. We implement the full-CNN architecture of the NeurASP framework to perform simultaneous classification of all the cells. As this architecture takes the whole image of the Sudoku as an input, we will refer to it as the *Whole-CNN* architecture.

***Shared CNN for cell-level classification***

One limitation of the *Whole-CNN* architecture is that it has to be trained with Sudoku images. This would be true for any generic perception-based constraint solving task, as the architecture can be trained with only task-specific data. Consequently, we might not have enough training data. For instance, from the Sudoku Assistant Android app, we could collect just 103 Sudoku images. The limited amount of training data hinders the learning of larger neural networks.

To address this limitation, we propose to do classification of each cell separately. In this setup, the classifier returns a 10-dimensional probability vector for each cell. There are several advantages to considering such *Cell-CNN* architecture. Firstly, in this approach, we train the model for each cell, so we have a larger number of training samples. For instance, from the 103 Sudoku images, we generate $103 \times 81$ training samples. We will discuss how we perform segmentation of a given Sudoku image in 81 cells without knowing the location of each cell in a separate paragraph. Secondly, it allows us to make use of *transfer learning* [44] more easily, by decomposing the whole image into multiple smaller-scale classification problems for which related datasets and trained neural networks are more likely to exist. In this case, we could use a *backbone* trained for digit classification on another dataset and then fine-tune it for the Sudoku images. Thirdly, the *Cell-CNN* architecture has a fewer number of trainable parameters than the *Whole-CNN*. As we leverage transfer learning, the need to learn the task of digit classification from scratch is eliminated.

#### Task-aware pre-processing

***Image segmentation with overlapped boundaries***

Training a *Cell-CNN* requires feeding each individual cell as training data. However, the input comes as a noisy image of the grid, wherein we do not know the exact location of the cell boundaries. We now discuss how to split a given, possibly noisy, Sudoku image into $9 \times 9$ cell-level images. The base approach is to split the input image into 81 blocks of equal height and width. However, natural images often exhibit slight rotations and imperfect cropping, resulting in the presence of borders. There is hence a misalignment expected between the segmented cells and the true objects. To broaden the horizon of the cell classifier, we investigate the added benefit of including some *overlaps* between the cells. To accomplish this, we first pad the image in such a way that we can extract 81 same-sized patches, all centered at the same spot as when no overlap is employed. That is, we can segment the image into cells in such a way that each cell’s patch overlaps a certain percentage with the neighboring cell patches. Our approach is motivated by the work in extracting overlapping patches from an original image, which is a well-studied technique in constructing OCR systems for handwritten word recognition [45].

***Data augmentation***

A *Cell-CNN* processes square regions of the initial input. For Visual Sudoku, each processed patch covers one cell and its neighboring pixels. The classifier thus learns to predict digits from raw images, which is a common task in computer vision [46]. This allows us to build the CNN with a pre-trained backbone, instead of training from the ground up. This also enables the use of data augmentation pipelines built specifically for the task the backbone was trained on, as provided by AutoAugment [47] for digit classification on raw images for example. Note that, such a data augmentation approach, is suitable only for *Cell-CNN* architecture. Applying a similar approach for *Whole-CNN* would require a CNN pre-trained on a visual task adjacent to our perception-based solving problem.

***Data imbalance*** In Visual Sudoku, the *blank* class has a higher prevalence, as the majority of cells in a Sudoku are blank, to be filled out by users. If left unchecked, this imbalance in labels induces a bias towards the majority class during training. One possible solution is to downsample instances of the most frequent classes while training. We evaluate three possible solutions to achieve a more balanced distribution while training: a) downsampling more frequent, b) upsampling infrequent instances, and c) considering a loss weighted with respect to the frequency of each label in the current mini-batch. Note that sampling-based methods assume a finer-grained level of inference, whereas the loss re-weighting method is applicable in any case.

We will evaluate these design choices and considerations when training the classifier in the experiments later.

### Calibration

The neural network performs the perception task and the probabilistic output of the network will be used by a CP solver. Hence, it is important that the probabilities returned by the neural network accurately represent the true likelihood of all the classes. However, due to several reasons, the probabilities returned by a neural network (especially a deep network) might not reflect the true underlying probabilities [48]. In machine learning, *calibration* is the process of modifying the predicted probabilities so that they match the expected distribution of probabilities for each class [48]. For example, a neural network that outputs a probability score of 0.3 for a given input should correctly classify inputs of similar labels 30% of the time. This property is important when considering a classifier within a broader autonomous system [49].

We will empirically assess the effect of calibration on our joint inference approach for perception-based constraint solving problems. Our framework reasons overall probability estimates $\{(P_\theta (y= blank |X), \ldots , P_\theta (y=p|X)\}\}$ and actively trades off the probability of a prediction of one image to the prediction of another image in its objective function. As such, our reasoning approach is directly impended by over or under-confident class probability estimations.

#### Confidence calibration

In a multi-class setting, for a given handwritten digit a neural probabilistic classifier computes a vector *z* containing raw scores called logits, for each class (i.e., a digit value), $z_k$ being the score assigned to class *k*. The SoftMax function is then applied to convert these logits into probabilities:

$$ \sigma _{\text {SoftMax}}\left( {\textbf {z}}_{k}, {\textbf {z}}\right) = \frac{\exp \left( {\textbf {z}}_{k}\right) }{\sum _{i} \exp \left( {\textbf {z}}_{i}\right) }. $$

such that $P_\theta (y=k|X) = \sigma _{\text {SoftMax}}\left( {\textbf {z}}_{k}, {\textbf {z}} \right)$ is the output of the neural network.

Post-hoc methods such as Platt scaling [50] aim at calibrating the probabilistic output of a pre-trained classifier. Guo et al. [48] describe three variants of Platt scaling in the multi-class setting. In *matrix scaling*, a weight matrix ${\textbf {W}}$ and a bias vector ${\textbf {b}}$ apply a linear transform to *logits*, the input vector of the softmax layer ${\textbf {z}}_i$ such that the calibrated probabilities become:

4 $$\begin{aligned} \widetilde{P}_\theta ^{\text {MatS}}(y_i=k|X_i)= \sigma _{\text {SoftMax}}\left( {\textbf {W}}_{\textbf {k}} {\textbf {z}}_{\textbf {k}}+{\textbf {b}}_{\textbf {k}}, {\textbf {W}} {\textbf {z}}+{\textbf {b}}\right) \end{aligned}$$

where **W** and **b** are parameters, learned by minimizing the Negative Log Likelihood loss on a validation set. *Vector scaling* applies the same linear transform, except that **W** is a diagonal matrix, that is, only the diagonal is non-zero. Finally, *Temperature scaling* considers a single scalar value *T* to calibrate the probability such that:

5 $$\begin{aligned} \widetilde{P}_\theta ^{\text {TempS}}(y_i=k|X_i) = \sigma _{\text {SoftMax}}\left( \dfrac{{\textbf {z}}_{k}}{T}, \dfrac{{\textbf {z}}}{T}\right) \end{aligned}$$

To *calibrate* the predictions, we train a model $f_{\theta ,{\textbf {W}},{\textbf {b}}}(X)$ where $\{(\widetilde{P}_\theta (y=1|X), \ldots , \widetilde{P}_\theta (y =p|X)\}\}$ is calibrated on a validation set $\mathcal {I}_{valid}=\{(X_i,y_i)\}$. More specifically, we perform calibration on top of a pre-trained neural network, so $\theta$ is pre-trained and the calibration learns the best **W** and **b** from the validation set $\mathcal {I}_{valid}$.

**Fig. 3:**
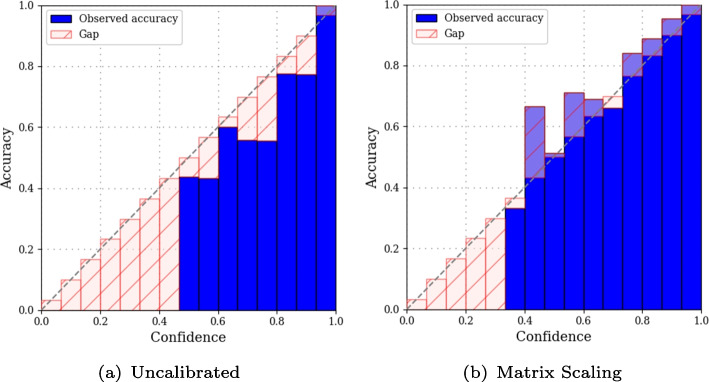
Confidence-reliability diagrams of a CNN on MNIST digits. Blue bars represent the observed accuracy within each bin, the red bars show the confidence-accuracy gap

#### Evaluation of calibration

There are multiple measures of calibration quality for probabilistic models. In the context of Perception-based Constraint Solving, the choice of calibration measure should reflect the goal of finding the correct solution from predicted probabilities.

***Expected Calibration Error (ECE)***

The ECE [51] approximates the difference in expectation between the confidence of a classifier, i.e. the probability assigned to the most likely class, and its accuracy. It is commonly estimated by binning predictions in equal-width bins, with respect to their confidence level. For each bin, $b_i$ is the set of indices of samples whose prediction confidence falls within the interval $(\frac{i-1}{M}, \frac{i}{M}]$ [48]. The ECE then is the average of the gap in accuracy with respect to the given confidence level for each bin, weighted by the number of samples

6 $$\begin{aligned} \text {ECE}(b) = \sum _{i=1}^{M} \frac{|b_i|}{n} | acc (b_i) - conf (b_i)| \end{aligned}$$

where *M* is the number of bins, $acc(b_i)$ is the fraction of true positives in bin *i*, and $conf(b_i)$ is the mean of probability scores in bin *i*. Confidence-reliability diagrams, as in Fig. 3, are used to visualize the ECE. In Fig. 3(a) the observed accuracy is below the average confidence in most bins. This figure illustrates that a CNN trained on MNIST [37] exhibits over-confidence. This tendency to over-confidence is commonly observed in neural networks that predict probabilities, trained by minimising cross-entropy (2) [49]. Figure 3(b) shows the effect matrix scaling, refining probabilities to bring the classifier closer to being perfeclty calibrated.

***Maximum Calibration Error (MCE)***

In [51], a variant of ECE is proposed which is more suitable for high-risks settings where minimizing the deviation between confidence and accuracy in the worst case becomes critical. If the ECE averages calibration errors across binned predictions, the MCE only considers the worst deviation.

7 $$\begin{aligned} \text {MCE}(b) = \max _{i \in \{1,\ldots ,M\}} | acc (b_i) - conf (b_i)| \end{aligned}$$

***Brier score***

In the context of probability calibration, the Brier score quantifies the deviation between confidence and accuracy of an estimator by calculating their mean-squared error.

8 $$\begin{aligned} \text {Brier}(b) = \sum _{i=1}^{M} \frac{|b_i|}{n} ( acc (b_i) - conf (b_i))^2 \end{aligned}$$

***Class-wise Expected Calibration Error (CW-ECE)***

In [49], the authors argue that the miscalibration of a single class is not explicitly reflected in the ECE. Indeed, it fails to capture the interactions between classes, as instances are binned in a one-versus-rest manner. Thus, they propose *classwise-ECE*, which measures the average confidence-accuracy gap across all single-class reliability diagrams

9 $$\begin{aligned} \text {classwise-ECE}(b) = \frac{1}{K} \sum _{k=1}^{K} \sum _{i=1}^{M} \frac{|b_{i,k}|}{n} | acc (b_{i,k})- conf (b_{i,k})| \end{aligned}$$

where *K* is the number of classes and $b_{i,k}$ is a set of indices of samples whose prediction confidence falls within the interval $(\frac{i-1}{M}, \frac{i}{M}]$, specifically for class *k*.

We will investigate the relationship between the choice of criterion for calibration and the solution quality experimentally, and show that calibrated predictions improve performances of our hybrid framework significantly in Section 7.

## Joint inference for full images by integrating classification and constraint solving

In the previous section, we describe the architecture of the neural network classifier, which performs perception. This section describes the CP modeling of perception-based constraint solving problems (PBCSP), with natural images as input.

### CP for perception-based constraint solving

Since the reasoning task is dependent on the perception task, we also have to model the interaction between the classifier and the CSP. To model this interaction, we next introduce *perception variables*, *channelling constraints*, and the objective function.

#### Perception variables

We introduce *perception variables* to decouple the modeling of the classifier and the modeling of the CSP. The perception variables, which we denote as $\mathcal{Y}$, are auxiliary decision variables. They map directly to the output of the neural network classifier, with their domain $\mathcal{D}(\mathcal{Y})$ being the set of all possible classes of the classifier.

***Example: visual Sudoku***

In the context of *Visual Sudoku*, the classifier identifies whether a cell is blank and if it is not blank, the number in that cell. So, the output of the classifier would be either a number between 1 and 9, or blank. The perception variables hence have domain $\{\texttt {blank},1,\dots ,9\}$. However, a solution to a Sudoku instance can only contain numbers between 1 and 9 without the option of being blank. Hence, the domain of Sudoku’s decision variables is the set $\{1,...,9\}$. The introduction of perception variables provides further modeling freedom; for example, as we will later see, a neural network could be trained to recognize both handwritten and printed digits, with different class labels for each, without changing the other decision variables.

#### Channelling constraints

Perception variables need to be related to decision variables through appropriate *channelling constraints*. This will typically be in the form of implication constraints of the style:

10 $$\begin{aligned} \text {channelling}(\mathcal {V},\mathcal{Y}) :=\llbracket \mathcal{Y} = k \rrbracket \Rightarrow \llbracket \mathcal {V}= v(k) \rrbracket \quad \text {for } v(k) \in \mathcal{D}(\mathcal {V}) \end{aligned}$$

The implication reads as follows: if perception variable $\mathcal{Y}$ is assigned class *k*, then decision variable $\mathcal {V}$ should be the assigned value *v*(*k*) of class *k*.

***Example: visual Sudoku***

For the visual Sudoku, channeling constraints between the perception variables $\mathcal{Y}_{rc}$ and decision variables $\mathcal {V}_{rc}$ are defined as follows:

11 $$\begin{aligned} \llbracket \mathcal{Y}_{rc} = k \rrbracket \Rightarrow \llbracket \mathcal {V}_{rc} = k\rrbracket \quad \text {for } k \in \{1,\ldots ,9\} \end{aligned}$$

Note that when $\mathcal{Y}_{rc}$ is blank, the value of the corresponding decision variable $\mathcal {V}_{rc}$ is not assigned, but is inferred by the CP solver.

In the next section, we formalize the problem of reasoning over constraints with a given visual input.

### Perception-based solving with natural images as input

$\texttt {PBCSP}(\mathcal {C}_{rules}, X, \theta )$ can be expressed as the following COP

12a $$\begin{aligned} \underset{\mathcal {V}, \mathcal{Y} }{\arg \min }  &   \mathcal {L}_{\theta }(X, \mathcal{Y}) \end{aligned}$$

12b $$\begin{aligned} \text {subject to}  &   \text {channelling}(\mathcal {V},\mathcal{Y}) \end{aligned}$$

12c $$\begin{aligned}  &   \mathcal {V}\in \text {CSP}(\mathcal {C}_{rules}) \end{aligned}$$

The goal is to find a value assignment of $\mathcal {V}$ and $\mathcal {Y}$ that minimizes the joint-inference error in the objective (12a). Note that we have two disjoint sets of variables: *perception variables* $\mathcal{Y}$ in the objective and regular *decision variables* $\mathcal {V}$ in constraints. They are linked through a set of channeling constraints in (12b). The cost function used to guide the solver depends on learned parameter $\theta$ from the perception layer; a CNN pre-trained on $\mathcal {I}_{train}$.

***Examples: visual Sudoku***

Formally, it can be described as $\texttt {PBCSP}(\mathcal {C}_{rules}, X, \theta )$ where $\mathcal {C}_{rules}$ defines the problem constraints, the input *X* is an image of $h \times w$ pixels in RGB space, and $\mathcal {I}_{train}=\{X_i, y_i\}_{i=1}^n$ is the training set, with $X_i \in \mathbb {R}^{h \times w}$ denoting images and $y_i \in \{\texttt {blank},1,\ldots , 9\}^{9 \times 9}$ their labels in the training set.

#### Joint inference

Joint inference describes processes through which capabilities of a learned estimator and a reasoning algorithm are combined to solve a decision problem [52]. In the past, it has been successfully applied to various Natural Language Processing tasks [53, 54].

Let $f_\theta$ be a probabilistic classifier and let $P_\theta (\mathcal{Y}_{rc} = k \ | X)$ be the predicted probability of $\mathcal{Y}_{rc}$ being of class *k*. A naive approach to applying joint inference for perception-based constraint solving is to consider, for each cell $\texttt{r,c}$, the predicted value *k* of maximum probability. As stated earlier, this approach lacks flexibility, as the solver cannot find a solution if the learned classifier is inaccurate.

A smarter approach is to make use of the full probability vector of each cell, so that the CP solver can reason over all probability distributions jointly. The satisfaction problem turns is transformed into an optimisation problem. The goal is to select the most probable value assignment to perception variables that satisfies all constraints and provides a solution to the CSP.

We assume that each perception variable is conditionally independent. Hence the likelihood of a joint assignment *S* to $\mathcal{Y}$ is

13 $$\begin{aligned} P_{\theta }(\mathcal{Y} = S) = \prod _{rc} P_\theta (\mathcal{Y}_{rc} = S_{rc} \ | X). \end{aligned}$$

The CP solver will *search* for an assignment $S_{rc}$ to the perception decision variables $\mathcal{Y}_{rc}$ for all *r* and *c*. Our goal is to select an assignment that has the highest likelihood. Note that the CP solver only returns assignments which adhere to the problem constraints $\mathcal {C}_{rules}$; so intuitively we select the most likely feasible assignment.

We will model this as a summation of element constraints. To do so, we transform the product the product in (13) into a sum by applying a logarithm and represent the log-likelihood in the following form:

14 $$\begin{aligned} \log P_{\theta }( \mathcal{Y} = S) = \sum _{rc} \log P_\theta (\mathcal{Y}_{rc} = S_{rc} \ | X). \end{aligned}$$

Furthermore, as the perception variable $\mathcal{Y}_{rc}$ can take exactly one value $K_{rc}$, we can write (14) as

15 $$\begin{aligned} \log P_{\theta }(\mathcal{Y} = S) =&\sum _{rc} \sum _{k \in \mathcal{D}(\mathcal{Y}_{rc})} \llbracket \mathcal{Y}_{rc} = k\rrbracket \log P_\theta (\mathcal{Y}_{rc} = k \ | X) \end{aligned}$$

where the indicator function $\llbracket \mathcal{Y}_{rc} = k \rrbracket$ returns 1 only if the decision variable $\mathcal{Y}_{rc}$ takes the value *k*. As our objective is to find an assignment with the maximum log-likelihood, we can set the objective of the CP solver to maximize $\log P_{\theta }(Y = K)$. Hence, the objective function $\mathcal {L}_{\theta }(X,\mathcal{Y})$ in () can be written in the following form:

16 $$\begin{aligned} \mathcal {L}_{\theta }^{\text {HCR}}(X,\mathcal{Y}) =&-\sum _{rc} \sum _{k \in \mathcal{D}(\mathcal{Y}_{rc})} \llbracket \mathcal{Y}_{rc} = k\rrbracket \log P_\theta (\mathcal{Y}_{rc} = k \ | X) \end{aligned}$$

Treating $\log P_\theta (\mathcal{Y}_{rc} = k \ | X)$ as a vector, note that the inner sum is easily modeled as an *element* constraint in CP.

#### Higher order knowledge exploitation

Second-order constraints are invariant properties that apply to all feasible solutions, but are not an explicit part of the problem description. They encode relational properties among variables of the problem. Thus, they provide additional useful information to solve it more efficiently. When performing joint inference for perception-based constraint solving, we can exploit them to further guide the search. However, exploiting these relationships during inference is challenging because they cannot be enforced through a classical CP search, or because they require deeper knowledge about the reasoning task, which usually emerges indirectly from first-order constraints, which here refer to the set $\mathcal {C}_{rules}$ of explicit rules.

Let $\mathcal {C}_{ higher-order }$ be a set of higher-order constraints on the ${{\,\textrm{CSP}\,}}$, expressed as

17a $$\begin{aligned} \not \exists \mathcal {V}^\prime :&~(\mathcal {V}, \mathcal{Y} ) \in \texttt {PBCSP}(\mathcal {C}_{ rules }, X, \theta ),&\end{aligned}$$

17b $$\begin{aligned}&~\mathcal {V}\ne \mathcal {V}^\prime ,&\end{aligned}$$

17c $$\begin{aligned}&~(\mathcal {V}^\prime , \mathcal{Y} ) \in \texttt {PBCSP} (\mathcal {C}_{ rules } X, \theta ) \end{aligned}$$

We start with finding the most likely assignment $(\mathcal {V}, \mathcal{Y} )$, as shown in (17a). The negated existential quantifier (17a) states that no other assignment $(\mathcal {V}^\prime , \mathcal{Y} )$ should exist for the decision variables $\mathcal {V}$ and $\mathcal{Y}$ that satisfies the set of constraints $\mathcal {C}_{rules}$. We can efficiently achieve higher-order reasoning by repeatedly checking if such a solution exists, and adding it as *nogood*, a forbidden assignment, to the set of constraints, before solving again. Such usages of blocking clauses and repeated solving are common approaches for handling second-order constraints [55].

***Example: Uniqueness property for visual Sudoku***

A valid Sudoku puzzle admits a unique solution for a set of givens. For traditional puzzles, this is the case by construction, as otherwise, a human solver would be faced with having to choose among two or more options, rather than reasoning up to a full solution. In our setting, however, the solver decides whether a cell is given or not through *perception variables*. Any such decision should lead the solver to a unique assignment of *decision variables*.

We present an instance of this approach in the conference article [17] for the uniqueness property. The described algorithm can be adapted to the current setting where we reason over the full image, by triggering the addition of *nogood* constraints whenever the current assignment of *perception variables* $\mathcal{Y}$ leads to multiple feasible assignments of *decision variables* $\mathcal {V}$. This is efficiently computed by checking the existence of more than one interpretation of $\mathcal {V}$ for a given $\mathcal{Y}$.

#### Scalability

The proposed joint-inference approach considers a modified problem where the solver reasons over (log)probabilities, and searches the optimal feasible assignment of decision variables with respect to the joint likelihood. This resulting problem is a COP of a larger feasible space to explore. In worst cases, the runtime soars above the thirty seconds mark if the discriminatory power of predicted probabilities is lacking. This latter situation is especially common when using a poorly-trained DNN. A simple way to mitigate this issue is to pre-train the neural network for more epochs (or different hyperparameters) and to apply confidence calibration as described in Section 4.2.

Given a well-trained classifier, we assume that the true value of each cell receives a relatively high probability score against other values in its domain. Hence, a straightforward scheme to further prune the search space is to define a threshold value $\tau \in [0,1]$ that discards values of lower probability. A similar strategy is to only consider *top-k* predictions for each cell during the search. Both approaches are common in tractable constraint solving [56]. Note that in case of erroneous human input or weak probabilistic classifier, all feasible solutions of $\texttt {PBCSP}(\mathcal {C}_{rules}, X, \theta )$ may have a low joint likelihood. Pruning the search space can then lead to the solver not being able to find any solution satisfying all constraints.

We will empirically investigate the effect of these pruning techniques on the overall solution quality in our experimental evaluations in Section 7.

## Handling human input for visual sudoku

The framework and its modules introduced in the previous sections operate under the strong assumption that the input image satisfies the problem constraints, e.g., it is a valid Sudoku. However, this assumption does not hold in many real-world applications, where the image may unintentionally or deliberately violate some of the constraints. For example, the input image can be entirely out-of-domain, or an image of a problem in which a user has entered incorrect information. Handling the former requires the perception module to learn to reject erroneous or non-relevant images [57, 58], which is beyond the scope of this study. In this section, our focus is on input images that contain (possibly erroenous) user input. We illustrate this application with images of Sudoku puzzles partially filled by humans.

**Fig. 4:**
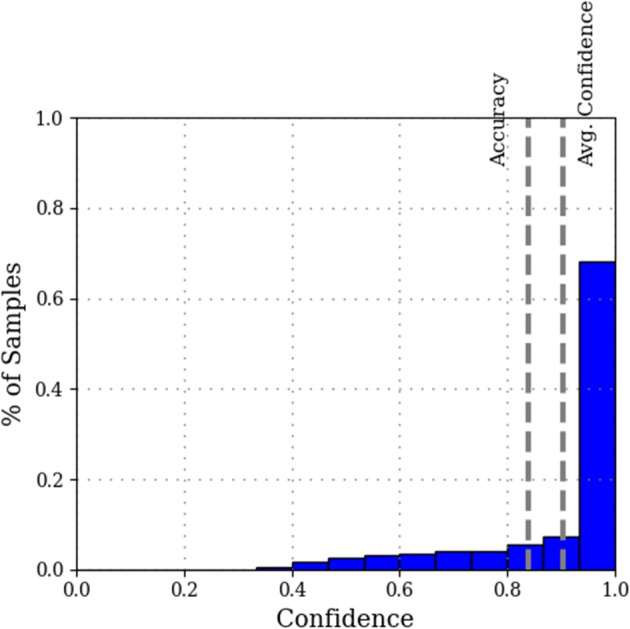
Confidence histogram of a *Cell-CNN* trained on data acquired through an app [19]. More than 60% of its predictions are strongly confident (above the 90% mark)

### Identifying erroneous input: font style classification

Let us consider the case of a Sudoku partially filled by a human user [40], as illustrated in Fig. 1. Such pen-and-paper Sudoku instances initially only contain blank cells and cells with printed digits. Hand-written digits are then manually added to certain cells by humans, making them human inputs on the grid. Moreover, the human inputs can be erroneous. This is illustrated in Fig. 1, where humans write a ‘2’ in the same column as a given ‘2’. In the considered setting, the erroneous input requires the solver to explore the next feasible value for that cell, which maximizes the likelihood.

For simplicity and ease of demonstration, let us assume a perfect classifier that assigns a probability of 100% to the right label. In this case, the approach proposed in Section 5 would only be able to find valid interpretations by correcting cells whose value leads to infeasibility. Since the classifier is perfect, all other values receive a probability of zero. This makes the solver equally likely to either correct the erroneous cell or alter another cell. In practical settings, DNN confidence follows a long-tailed distribution, as shown in Fig. 4. Figure 4 displays that most of its predictions are confident, with probabilities exceeding 90%. The high probability values initially lead to a high-likelihood assignment of perception variables. In the considered setting, when erroneous input is encountered, the solver outputs the best feasible value that maximizes the likelihood. However, there is no assurance that it would correct the erroneous cell. Hence, the first step towards correcting such potential mistakes made by human users, is to identify hand-written cells. More broadly, in a generic perception based constrained solving problem, it entails identifying different types of input.

To build a classifier that predicts $P_\theta (\mathcal{Y}_{rc} = \texttt {printed} \ | X)$, we can train the same neural network for both digit value recognition and font style classification tasks, at the same time. To do so, we have to modify its output layer accordingly and make font-style labels available during training. To this end, we consider two approaches for modifying the *Cell-CNN* architecture. One approach would be to increase the number of labels, i.e., labels for printed as well as handwritten for all the 9 digits, resulting in 19 classes (blank, and for each digit, 2 labels—printed and handwritten). The second approach considers a multi-task learning setting where the heads of the classifier specialize in digit and font classification respectively [59]. We also point out that printed digits are inherently more uniform across images, whereas the same hand-written value may vary drastically across Sudokus, depending on the writing style of the human, the type of pen used and many such things. How we build and train CNNs, taking these aspects into consideration, is described in Section 7.7.1.

### Reasoning in the presence of erroneous input

The next step after identifying different types of input is to enhance our solver with the ability to reason about different types of input. In this respect, we propose two approaches.

#### Modifying the cost function for erroneous input

The first approach we propose modifies the cost function introduced earlier in (16).

As the hand-written digits are at the risk of being erroneous, we want the CP solver to assign lower emphasis on perception variables interpreted as $\texttt {handwritten}$.

So, we modify (16) in the following form to take into account the font style of the input:

18 $$\begin{aligned} \mathcal {L}_{\alpha , \beta , \gamma , \theta }(X,\mathcal{Y})&= - \sum _{rc}\biggl (\gamma \log P_\theta (\mathcal{Y}_{rc}=\texttt {blank} \mid X) \nonumber \\&+ \alpha \sum _{k\ \in \ \{1, \ldots , 9\} } \llbracket \mathcal{Y}_{rc} = k \rrbracket \log P_\theta (\mathcal{Y}_{rc} =k \wedge \mathcal{Y}_{rc}=\texttt {printed} \mid X) \nonumber \\&+ \beta \sum _{k\ \in \ \{1, \ldots , 9\} } \llbracket \mathcal{Y}_{rc} = k \rrbracket \log P_\theta (\mathcal{Y}_{rc} =k \wedge \mathcal{Y}_{rc}=\texttt {handwritten}\mid X)\biggr ) \end{aligned}$$

In (18) the non-negative coefficients $\alpha , \beta$, and $\gamma$ represent weights assigned to blank, $\texttt {printed}$ and handwritten respectively. We can further reduce (18) to a single hyper-parameter $\alpha$, by aggregating the probability of blank and $\texttt {printed}$.

19 $$\begin{aligned}&\mathcal {L}_{\theta ,\alpha }^{\text {multi}}(X,\mathcal{Y})\nonumber \\  &= - \sum _{rc}\biggl (\nonumber (1-\alpha )\sum _{k\ \in \ \{1, \ldots , 9\} } \llbracket \mathcal{Y}_{rc} = k \rrbracket \log P_\theta (\mathcal{Y}_{rc} =k \wedge \mathcal{Y}_{rc} = \texttt {handwritten} \mid X) \nonumber \\&\!+\! \alpha \sum _{k\ \!\in \! \ \{\texttt {blank},1, \ldots , 9\} } \llbracket \mathcal{Y}_{rc} \!=\! k \rrbracket \log P_\theta (\mathcal{Y}_{rc} \!=\!k \wedge (\mathcal{Y}_{rc} \!=\! \texttt {printed} \vee \mathcal{Y}_{rc} = \texttt {blank} )\mid X) \biggr ) \end{aligned}$$

The coefficient, $\alpha \in [0,1]$, explicitly controls the influence of digits recognized as hand-written on the search for a solution. The case where $\alpha =1$ denotes a setting where the solver solely relies on cells identified as blank or containing printed values to find a feasible solution, thus avoiding noisier hand-written input.

#### Modifying the constraints for erroneous input

The objective function defined in (19) sets the solver to assign low emphasis on hand-written digits, recognizing that they might be potential sources of human mistakes. The high-level motivation for incorporating erroneous input information into the constraints, described next, is to solve the problem despite the presence of a constraint violation in the input. In our example, we want to solver to alter the digit ‘2’ written by human users by completely ignoring what is written in that cell.

***Prior probability of input error***

Let us consider the case of a Sudoku partially filled by a human player, as illustrated in Fig. 1(b), with some mistakes (here, the ’2’ written in the same column as a given 2). Let us also consider a probabilistic classifier whose confidence distribution is depicted in Fig. 4. Most of its predictions are confident over 90%, which would initially result in a high-likelihood assignment of perception variables. In the considered setting, the erroneous input requires the solver to consider the next best feasible value for that cell according to our confident classifier. This results in the solver making trade-offs between low-scored values to find a low-likelihood puzzle interpretation.

Rather than ignoring individual digits written by the user, we want to allow the solver to consider the nature of each cell’s input, by integrating the erroneous input information into the constraints.

To implement this we add wildcard in the the domain of perception variables. This extends the domain of the perception variable as shown below:

20 $$\begin{aligned} \mathcal{D}(\mathcal{Y}_{rc}) = \{\texttt {blank},~1,\ldots ,~9,~\texttt {wildcard}\} \quad \forall \mathcal{Y}_{rc} \in Y \end{aligned}$$

We point out that both wildcard and blank share the same purpose for the perception variable. Namely, they are placeholders, indicating that the value of the corresponding decision variable $\mathcal {V}_{rc}$ should be derived by the solver, reasoning over problem constraints, and not from machine learning predictions. By selecting this option, we enable the solver to discard the predicted values of cells classified as hand-written. Instead, the solver derives its value by reasoning over problem constraints.

***Property-aware prior***

In order to decide which cell(s) will be assigned as wildcard, we modify the probability distribution of each cell by assuming a prior probability of user mistake. We propose the following formulation for the prior probability of user mistakes, utilizing the output of the perception module:

21 $$\begin{aligned} P_\theta (\mathcal{Y}_{rc} = \texttt {wildcard} \ |~ X) = \gamma _e P_\theta (\mathcal{Y}_{rc} = \texttt {handwritten} \wedge \mathcal{Y}_{rc} \ne \texttt {blank} \ |~X) \end{aligned}$$

The intuition behind the formulation in (21) is that the probability of a cell being a wildcard is proportional to its likelihood of being identified as hand-written. Here, $\gamma _e$ is a hyperparameter that explicitly controls the likelihood of a cell being a wildcard, based on whether the cell is classified as hand-written or printed. The setting $\gamma _e = 0$ corresponds to the extreme case of a null prior, which does not allow assigning wildcard to any perception variable. This approach allows for a more refined prior that takes into account each cells’ input nature. We will refer to this scheme as style-aware prior.

***Feasibility***

As described in Section 5.2.1, the symbolic search for an optimal solution is guided by the joint probability distribution over the entire grid. In this context, the prior probability of the input error, functions as a threshold. Specifically, the solver will exhibit a preference for choosing the wildcard over values with likelihood scores below the prior. A good threshold value should rank the wildcard option high or low respectively for cells with hand-written or printed digits. This threshold can also be strictly enforced by constraining the solver to disallow values whose probability scores fall below the specified prior. The channeling constraint with property-aware prior, $\text {channelling}(\mathcal {V}, \mathcal{Y}, \theta )$, can be formally described as below:

22a $$\begin{aligned}&P_\theta (\mathcal{Y}_{rc} \!=\! \texttt {wildcard} \ \mid X) > P_\theta (\mathcal{Y}_{rc} \!=\!k \mid X) \quad \forall k \!\in \! \{1,\ldots ,9\} \Rightarrow \llbracket \mathcal{Y}_{rc} \!=\! \texttt {wildcard} \rrbracket , \end{aligned}$$

22b $$\begin{aligned}&\llbracket \mathcal{Y}_{rc} = k \rrbracket \Rightarrow \llbracket \mathcal {V}_{rc} = v\rrbracket \quad \; \forall \; k \in \{1,\ldots ,9\} \end{aligned}$$

## Experiments

In this section, we present an empirical study that explores various design choices in building a hybrid perception and reasoning system for solving visual constraint satisfaction problems. The main research problem addressed here is providing a robust joint inference approach for perception based constraint solving. We aim to demonstrate its capability of correctly solving visual CSP instances, even when the input image may contain constraint violations. Our objective is to harness the power of CNNs as a perception layer to interpret user-provided images, combined with a constraint solver as a reasoning layer, to achieve accurate and efficient solutions.

To address this challenge, we formulate the following research questions:

***RQ1***.: What perception-level considerations are taken into account while training the CNN to improve the predictions?
***RQ2***.: Does leveraging higher-order knowledge improve joint inference, and can state-of-the-art approaches be enhanced with the joint inference approach?
***RQ3***.: To what extent does utilizing a calibrated model enhance joint inference?
***RQ4***.: Can the incorporation of prior probabilities of input errors enable the detection and correction of user mistakes in pen-and-paper Sudokus?

We will conduct a series of numerical experiments to evaluate our approaches on several datasets. All machine learning models and algorithms were implemented in the *lightning* environment [60], with PyTorch 1.10 [61]. All constraint reasoning was implemented in the *CPMpy* 0.9.13 [62] modeling environment, using the CP-SAT solver of OR-Tools 9.3.10497 [63]. For each experiment, we report results out of five cross-validation rounds with different seeds.

**Fig. 5:**
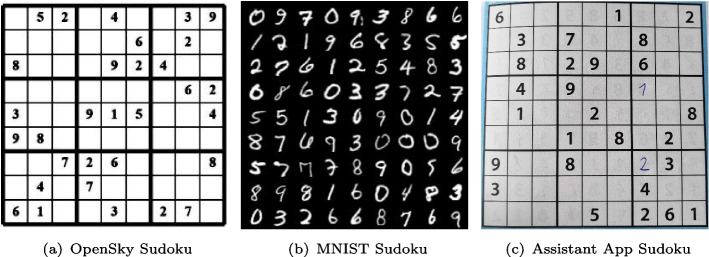
Samples from the three visual Sudoku datasets

### Sudoku datsets

Throughout this section, some experiments will consider multiple datasets of varying perception difficulties, to highlight the capacity of our joint inference approach to work with different CNN designs and choices of CP modeling [64]. Next, we describe the three datasets.

***OpenSky Sudoku generator***

This dataset contains 100 image-label pairs, extracted from the OpenSky Sudoku generator^1^, as described in [34]. As shown in Fig. 5(a), these images are perfectly aligned and centered around the puzzle, with no artifacts. Hence, we expect the perception tasks to be easy in this dataset.

***MNIST visual Sudoku***

The dataset is derived from the work of SATNet [13]. This dataset contains 10,000 numerical Sudoku instances, sampling a corresponding image from MNIST for each cell to obtain the visual reasoning task. The previous publications [13, 14, 17] consider the location of each cell to be known beforehand and take an image of one cell as an input. However, the focus of our work is to solve a Sudoku from the image of the whole Sudoku. Hence, we merge 81 cell images to form one global image of the puzzle. One sample of such reconstructed Sudoku is shown in Fig. 5(b). In this dataset, we fill the blank cells represented by ‘0’.

Additionally, we assume that all the constraints of our puzzle are known, and rely on a CP solver for joint inference. This differs from SATNet [13], which proposes a differentiable constraint satisfaction layer that i) learns the rules from noisy input, ii) assumes known locations of the initial clues, iii) relies on a learned solver for inference. The perception task in this dataset is slightly more difficult than in the OpenSky dataset, as the cells contain MNIST hand-written digits. Contrary to previous publications

***Sudoku assistant Android app***

The Sudoku Assistant [19] stores and processes pen-and-papers Sudokus scanned with users’ smartphone. This dataset is the most challenging to work with as varying lighting conditions, misaligned grids, blur, and other visual artifacts make the perception task harder. We used the app to build and label a dataset of 103 images. One sample of this dataset is shown in Fig. 5(c). We remark that, in this case, a Sudoku may contain digits written by humans. To make a distinction between hand-written and printed digits, each instance has two sets of labels: a label on the cell value (which can be blank or a number between 1 and 9) and a label on the font style (which can be either printed or hand-written). These two sets of labels are combined to form a single set of 19 labels, for tasks that require both digit recognition and font classification.

### Evaluation criteria

In order to test and compare the performances of different frameworks, we have the following evaluation metrics (adopted from our conference paper [17]):

*Cell accuracy:* It measures the proportion of cells correctly classified by the neural network classifier before the reasoning module. To evaluate this metric, we take the argmax of the probabilistic output of each cell and assign it as the predicted label and then compare it with the true label.

*Grid accuracy:* It measures the proportion of Sudoku instances whose cells are jointly classified correctly by the neural network.

*Cell accuracy (reasoning):* It measures the proportion of cells matching the true label after joint inference. To evaluate this metric, we consider the assignment of *perception variables* $\mathcal{Y}$ given by the solver for each cell and then compare it with the true classification label.

*Grid accuracy (reasoning):* It measures the proportion of Sudoku instances whose solution, after joint inference, matches with the true solution. If the labels of all of the 81 cells of a Sudoku instance are identical to the true labels, we consider the instance to be correct, otherwise, it is considered to be incorrect. Let $S^\star = CSP(\mathcal {C}_{rules})$ be the ground truth for a given image-label pair (*X*, *y*), and $(\hat{V}, \hat{Y}) =$ $\texttt {PBCSP}(\mathcal {C}_{rules}, X, \theta )$   the assignment obtained by joint inference. In a correct solution, $\hat{V}$ solves the problem instance, that is, $\hat{V} \equiv S^\star$.

### RQ1: training considerations for perception layer

This section outlines the considerations we take into account when training a CNN as a perception module. These considerations encompass deciding between predictions at cell or grid levels, addressing data imbalances through upsampling and downsampling, and the integration of pre-trained models. Recall that in Section 4.1 we introduced two levels of inference for our perception module, resulting in two distinct categories of CNN architectures: *Whole-CNN* and *Cell-CNN*. The former operates at the full-image level, processing the entire input image as a whole, while the latter operates at a local level, analyzing cells or smaller patches within the image independently.

***Data imbalance***

All Sudoku datasets considered are imbalanced, as blank cells are over-represented with respect to other labels. Thus we study the impact of three different data imbalance policies on classifier accuracy, namely downsampling the majority class, upsampling minority classes and re-weighting training instances with the inverse of their frequency in the current batch (WeightedCE). Note that the latter is applicable regardless of the CNN architecture, while resampling methods require operating at the cell level. Table 1 shows a positive effect of imbalance handling policies for both CNN architectures. Resampling improves the *Cell-CNN* accuracy from 91.84% to 92.78% on the Sudoku Assistant dataset. The impact is less pronounced on other datasets, as the baseline accuracy was already high.

***Data efficiency***

**Fig. 6:**
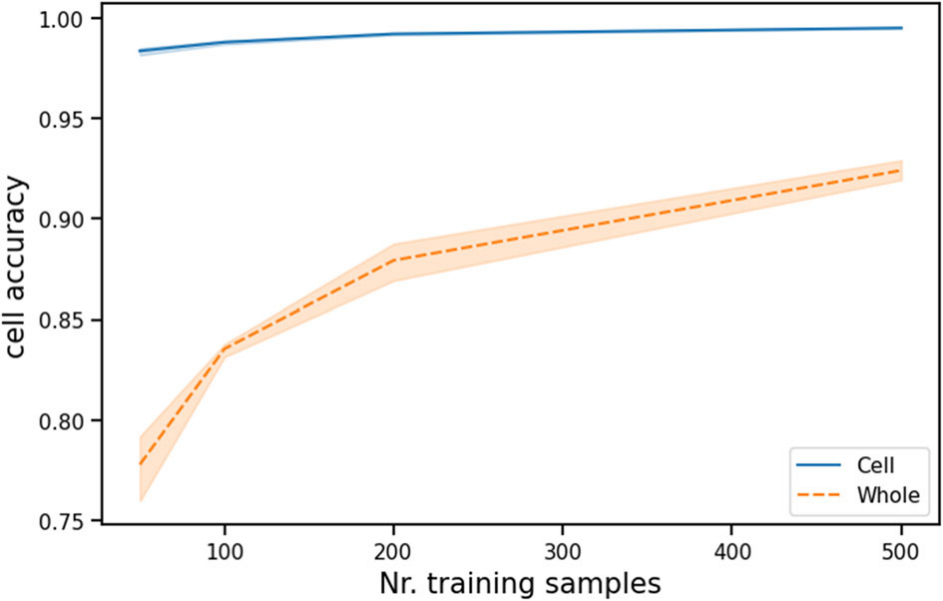
Cell accuracy over different amount of training samples, on the test set of MNIST data

*Cell-CNN* is more data efficient, as each image in the train set is turned into 81 training samples. This is highlighted in Fig. 6, where the accuracy of the cell-level architecture remains stable regardless of the number of training instances used. From the figure, we conclude that*Whole-CNN*requires more data to reach similar performance.

***Pre-trained backbone***

**Table 1:** CNN accuracies when trained with various imbalance policies

		Cell accuracy			
Dataset	Arch	None	WeightedCE	Upsampling	Downsampling
OpenSky	Cell	99.90%±0.13%	99.95%±0.07%	100.00%±0.00%	100.00%±0.00%
	Whole	99.76%±0.08%	99.68%±0.11%	–	–
MNIST-1000	Cell	98.25%±0.23%	98.26%±0.15%	98.32%±0.20%	97.98%±0.11%
	Whole	85.62%±16.94%	93.45%±0.88%	–	–
Assistant	Cell	91.84%±0.54%	91.37%±0.35%	92.78%±0.40%	91.62%±0.85%
	Whole	71.18%±9.07%	74.10%±1.62%	–	–

We study the applicability and benefits of transfer learning in our framework. For this experiment, we use a pre-trained layer as a feature extractor, followed by a digit classifier. In Table 2 and the subsequent tables, we emphasize models with the highest grid or cell accuracy. The outcome, presented in Table 2, displays two training scenarios: one with disabled gradient updates for backbone layers and another with enabled updates, where the latter requires lengthier training due to a larger number of trainable parameters. See the appendix for a description of each considered neural network architecture. Notably, the *Cell-CNN* outperforms others in accuracy, pertaining to both its data-efficient nature, as validated earlier, and the quality of considered backbones. These layers are pre-trained on single-output classification tasks, which may not align with the multioutput nature of digit recognition in the visual sudoku context. Remarkably, the backbone pre-trained on SVHN raises accuracy to 98.40% on the Assistant dataset. Moreover, even when the backbone’s parameters remain static during training, fine-tuning only its output layer still leads to *Cell-CNN* surpassing their *Whole-CNN* counterparts. Reaching a similar boost in performance for the *Whole-CNN* architecture would require access to parameters of a CNN specialized in grid recognition, as presented in [65].

**Table 2:** Accuracy CNNs built with different pre-trained backbones, trained with imbalance policies, and evaluated on test data from the assistant dataset

		Cell accuracy	
Level	Architecture (Dataset)	All weights	Output layer weights
Cell	Baseline *Cell-CNN*	92.78%±0.40%	–
	LeNet (MNIST)	92.50%±0.30%	65.60%±33.80%
	Resnet18 (ImageNet1K)	96.10%±0.30%	84.50%±0.80%
	**VGG16 (SVHN)**	**98.40%±0.30%**	**97.90%±0.50%**
Whole	Baseline *Whole-CNN*	74.10%±1.62%	–
	Resnet18 (ImageNet1k)	51.60%±3.10%	63.60%±2.00%
	Resnet50 (ImageNet1k)	54.20%±2.60%	63.00%±2.40%

For each architecture, *Output layer weights* indicates that only the output layer is trained on our dataset, while *All weights* indicates that all CNN weights are updated during backpropagation

The highest accuracy values are shown in bold for each case

**Table 3:** Performance of joint inference approach on test set, on all three datasets

Joint		Grid accuracy	Cell accuracy		Inference
inference	Architecture	(reasoning)	(reasoning)	Unsolved	time (sec.)
OpenSky					
baseline	LeNet	100.00%	100.00%	0.00%	0.038
hybrid	LeNet	100.00%	100.00%	0.00%	0.698
higher-order	LeNet	100.00%	100.00%	0.00%	0.748
MNIST					
baseline	LeNet	73.00%±0.00%	99.56%±0.00%	26.33%±0.00%	0.011±0.001
hybrid	LeNet	99.17%±0.24%	99.70%±0.07%	0%±0%	0.776±0.084
higher-order	LeNet	99.40%±0.43%	99.94%±0.02%	0%±0%	1.81±0.02
Assistant App					
baseline	VGG16	77.42%±3.23%	81.27%±0.85%	16.77%±1.44%	0.296±0.017
hybrid	VGG16	93.25%±2.64%	94.92%±3.04%	0%±0%	1.707±0.37
higher-order	VGG16	94.84%±1.77%	97.03%±1.73%	0%±0%	2.926±0.187

All *Cell-CNN* architectures are pre-trained with data augmentation and imbalance handling

### RQ2: joint inference

In this section, we study the effectiveness of several reasoning layer approaches for perception-based constraint solving. Specifically, we compare the rate of test instances correctly solved by reasoning over the joint probability distribution over all cells – denoted as hybrid – with or without higher-order knowledge – denoted as higher-order – against the simpler approach of solving over the argmax prediction of each cell, denoted as baseline.

***Solving approaches***

The results presented in Table 3 underline the limitations of the baseline approach. Even with an accurate classifier, the simple method of assigning the value of the highest probability mass to each cell leads to infeasible problem instances. Across all three datasets, conducting joint inference over the probability distribution space offers dual advantages: 1) it always provides a feasible solution and 2) enhances the rate of correctly solved instances. Further leveraging higher-order knowledge enhances these benefits, albeit at the expense of additional solver runtime.

As expected from previous experiments, not much information stems from results on OpenSky data, where the only difference between approaches is in solving time. Moving to the MNIST dataset, while all approaches use the same $99.54\%$-accuracy calibrated *Cell-CNN*, both hybrid and higher-order approaches improve grid accuracy by $20\%$ over that of the baseline. The latter fails to find any feasible solution for $26.33\%$ of all test instances. Similarly, on the Assistant App data, the baseline approach only solves correctly $77.42\%$ of test instances, while $16.77\%$ remain unsolvable within the specified time limit. This is increased to $94.84\%$ with higher-order joint inference, while the rate of unsolved test instances drops to zero, leaving $5.16\%$ instances for which the solver output does not match the known solution. Fortunately, the high cell accuracy after reasoning indicates an important overlap of matching cells between solver output and ground truth, even among non correct solution. Indeed, a cell accuracy of $99.2\%$ after reasoning accounts for only 21 out of 2511 mis-valued cells.

***Performance/Runtime tradeoff***

**Fig. 7:**
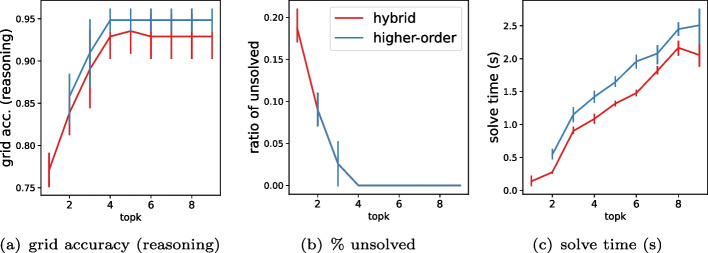
Comparison of constraint solving approaches, with varying *top-k* constraints, on Sudoku Assistant data. From left to right, figures illustrate the sensitivity of grid accuracy after reasoning, the rate of unfeasible instances and the average solve time, with respect to the hyperparameter *k*

Figure 7 shows the effect of considering *top-k* most likely values for each perception variable during the search. The system’s accuracy decreases at lower *k* levels, and the rate of unsolved instances surges, but the solving time decreases drastically. The *top-k* constraints allow for explicit control of the efficiency-performance trade-off, making hybrid probabilistic reasoning approaches more tractable if properly tuned. More specifically, on Assistant App data, using a VGG16 backbone CNN, considering the *top-4* value from each perception variable domain provides performance on par with reasoning on the full domain, with a lower solving time.

***Correcting effect on weaker classifiers***

Figure 7 also highlights the benefit of reasoning with higher-order constraints. In the context of the Visual Sudoku, reasoning with the uniqueness property leads to better performance even when only considering *top-k* predictions, as it more efficiently prunes away more erroneous assignments of perception variables. This allows the system to recover – up to an extent – from finding incorrect feasible solutions of high likelihood.

**Table 4:** Spearman correlation coefficient characterizing the relationship between calibration measures and grid accuracy after reasoning (with related *p*-values), computed over 40 samples across all datasets

ECE	Classwise-ECE	Brier	MCE	NLL
$-65.29\% (0.00\%)^\star$	$-64.56\% (0.00\%)^\star$	${\textbf {-75.30\%}} {\textbf {(0.00\%)}}^\star$	$27.43\% (9.10\%)$	$-59.35\% (0.01\%)^\star$

Boldness denotes strong correlations. The symbol $\star$ denotes statistically significant correlations (at 1% level of significance)

### RQ3: calibration

The key difference between standard supervised learning and our neurosymbolic setting is the presence of a downstream reasoning task taking predictions as input. This section evaluates the effectiveness of further exploiting this knowledge to improve performance of the pre-trained perception module.

The CP solver conducts joint inference by combining probabilistic predictions with constraint reasoning. This requires well-calibrated probabilities from the neural network, because search space exploration directly depends on estimated probabilities. This section studies the benefits of considering calibrated classifiers for perception-based constraint solving.

***Relationship between calibration evaluation method and performance***

We compute samples were obtained by calibrating pre-trained *Cell-CNN* on each dataset and then used the hybrid solver to compute grid accuracy after reasoning. This process was repeated five times with different seeds and calibration methods. We omitted samples from the OpenSky dataset because Cell-CNNs would always achieve 100% accuracy.

We calibrate pre-trained Cell-CNN on each dataset, then use Hybrid constraint solving to find a solution and compute the *grid accuracy (after reasoning)* for this trial. The process is repeated five times with different seeds. We omitted the Opensky dataset because Cell-CNNs would always get 100% cell accuracy, which does not provide meaningful information. Table 4 displays a Spearman correlation coefficient and the statistical significance of a correlation between calibration measures and solving performance measures, across datasets. For each metric, a negative coefficient implies that if this metric decreases, the ratio of correct solutions found by the solver increases. A positive coefficient implies that both would increase or decrease according to one another. A value of 100% or -100% indicates a strong correlation, while 0% denotes an absence of correlation.

All calibration measures exhibit strong negative correlations with *grid accuracy (after reasoning)*, except for MCE as defined in (7). This metric aims to capture the impact of miscalibrated prediction in a downstream high-risk decision problem. Solving a visual puzzle with Hybrid probabilistic reasoning alleviates the negative impact of prediction errors. Brier score appears to have the strongest correlation with solving performance.

***Comparison of Platt scaling calibration methods***

Table 5 shows a comparison of Platt’s scaling variants in their impact on the downstream reasoning task. We consider Temperature, Vector, and Matrix calibration methods as introduced in (4) and (5). For each dataset, the same pre-trained *Cell-CNN* is calibrated (not for the uncal baseline) with each considered method. The Opensky dataset makes the comparison challenging as all calibration methods lead to $100\%$ grid accuracy. On MNIST, the baseline CNN appears already well calibrated, with none of the methods resulting in a lower Brier score. Both Temperature and Vector scaling contribute to improving the grid accuracy, the latter being more effective. Then on the Assistant dataset, Matrix scaling emerges as the most effective, yielding grid accuracy increase while exhibiting lower variance. Note that Vector scaling here leads to a lower average grid accuracy, but with a higher variance. Thus indicating that it outperforms the winning variant on some trials. In conclusion, one of Matrix and Vector scaling surpasses Temperature scaling, when it comes to performances of the downstream reasoning task. This aligns with previous findings [17]. Platt’s scaling method tunes a layer that controls the probability distribution estimated by the CNN. Temperature scaling does so by considering a single value, controlling the smoothness of the output distribution. On the other hand, Matrix and Vector scaling offer a finer-grained smoothing, by exploiting correlations between labels.

**Table 5:** Comparison of calibration methods on all datasets, against the uncalibrated (Uncal.) baseline

Calibration	Brier	Grid accuracy (reasoning)
OpenSky		
Uncal	**0.00%±0.00%**	**100.00%±0.00%**
Temperature	**0.00%±0.00%**	**100.00%±0.00%**
Vector	**0.00%±0.00%**	**100.00%±0.00%**
Matrix	**0.00%±0.00%**	**100.00%±0.00%**
MNIST		
Uncal	**0.07%±0.01%**	98.93%±0.28%
Temperature	**0.07%±0.01%**	99.00%±0.27%
Vector	**0.07%±0.01%**	**99.33%±0.24%**
Matrix	0.08%±0.01%	98.73%±1.42%
Assistant		
Uncal	0.24%±0.04%	93.55%±3.23%
Temperature	0.24%±0.04%	93.55%±3.23%
Vector	0.52%±0.54%	90.32%±6.03%
Matrix	**0.18%±0.02%**	**94.84%±1.77%**

For each measurement, mean and standard deviation over 5 trials are reported. The bold fonts denote the best method for the corresponding measurement

### Comparison with related approaches

We now assess our approach by evaluating its use within other frameworks, namely NeurASP [34] and NASR [42].

#### Comparison with NeurASP

The NeurASP framework [34] also performs probabilistic inference to find the maximum a posteriori value assignment of variables, which satisfies the constraints. Although NeurASP [34] also allows for end-to-end learning through the constraints, it is strictly used as a reasoning module in this experiment.

Regarding joint inference, the differences between NeurASP and our framework revolve around the encoding of neural network probabilities and the inference mechanism of each underlying constraint-solving technology. Constraints in NeurASP are declared through a logic program. The probabilistic output of the neural network is encoded through the *neural atom*. In the logic program, they act as syntactic sugar that matches each probabilistic output of the neural network to an identifier atom. This is similar to our usage of *perception variables*, whose domain corresponds to possible outcomes predicted by the CNN. However, we explicitly encode those probabilities through the cost function of our CP model, whereas in NeurASP, they occur as probability distributions over facts and rules of the logic program. The ASP solver can efficiently enumerate *answer sets* which maximizes the probability of all neural atoms. In our CP-based approach, the predicted probabilities occur explicitly in the cost function used by the branch-and-bound algorithm to guide the search. This provides additional control over the information used by the solver during the search, as proposed in Section 6.2.1.

Regarding runtime, the results depicted in Table 6 indicate that, when using predictions out of the same CNN, joint inference in NeurASP is significantly faster, while performing on par with our Hybrid approach. However, NeurASP is outperformed by our Higher-order approach. Note that a combination of ASP and Neural Networks can benefit from the advanced solving schemes introduced in this paper, which is a promising avenue for future research.

**Table 6:** Comparison of solving approaches with related work, on Sudoku assistant data

Method	Grid accuracy	Cell accuracy		Inference
	(reasoning)	(reasoning)	Unsolved	time (sec.)
NASR-baseline	21.29%±1.77%	44.13%±0.26%	78.71%±1.77%	0.298±0.012
NASR-higher-order	43.55%±3.23%	43.88%±0.28%	0%±0%	61.186±23.169
NASR-hybrid	66.45%±7.77%	44.13%±0.24%	0%±0%	20.203±0.941
NeurASP	94.19%±2.70%	97.85%±1.08%	0%±0%	0.678±0.008
Hybrid	94.84%±1.77%	98.08%±0.79%	0%±0%	2.049±0.041
Higher-order	**96.13%±1.44%**	99.23%±0.13%	0%±0%	1.862±0.110

All hybrid solvers use the same 5-layers VGG16 *Cell-CNN*, pretrained on SVHN, calibrated with Matrix scaling, with data imbalance handling

The highest grid accuracy value is shown in bold

#### Joint inference with NASR

Let $\hat{s} = \text {SolverNN}_{\omega _1}(\hat{y})$ be the probabilistic solution predicted by the SolverNN transformer, with $\hat{s} \in \mathbb {R}^{81 \times 9}$ defining a probability distribution over $\{1, \ldots , 9\}^{81}$ and $\hat{m} = \text {MaskNN}_{\omega _2}(\hat{s})$ be the probabilistic mask predicted by the MaskNN transformer, with $\hat{m} \in \mathbb {R}^{81}$. The work in [42] considers a non-probabilistic symbolic solver encompassing rules of the Sudoku, in Prolog [66], such that

23 $$\begin{aligned} \mathcal {V}^{\text {NASR}} = {{\,\textrm{CSP}\,}}(\mathcal {C}_{rules} \wedge \mathcal {C}_{given}^{\text {NASR}}) \end{aligned}$$

where $\mathcal {C}_{given}^{NASR}$ is the set of constraints that assign neural network output to cell values, such that

24 $$\begin{aligned} \mathcal {C}_{given}^{\text {NASR}} := \forall ~\texttt{r,c}: \mathcal {V}_\texttt{r,c} \in \arg \max \hat{s}_\texttt{r,c} \cdot \hat{m}_\texttt{r,c} \end{aligned}$$

Equation (24) merges the SolverNN and MaskNN output into a probability matrix $\hat{Z} = \hat{m} \odot \hat{s} \in \mathbb {R}^{81\times 9}$, were $\odot$ stands for the element-wise multiplication operation. The symbolic solver takes as input the argmax value at each indices pair ($\texttt{r,c})$. Note that because NASR is trained on predicting the solution, its output domain does not account for blank cells. We can easily convert $\hat{Z}$ to a format suitable for our framework by padding its second dimension with a vector of zeros, accounting for the probability score of the $\texttt {blank}$ value. This turns NASR output into $\tilde{Z} \in \mathbb {R}^{81\times 10}$. This construction of $\tilde{Z}$ explains the low cell accuracy after reasoning observed in Table 6 for NASR-based approach. Since NASR is not trained to predict the empty label, the solver will rarely assign *empty* value to its perception variables.

***Experiment setup***

We compare performances of our solving approaches when applied to the output of NASR. Namely, we transform the perception module output by passing it through NASR’s SolverNN and MaskNN transformers, before giving it to any solver. In this experiment, we used off-shelves SolverNN and MaskNN, pretrained on the Big Kaggle dataset from [42], without any RL fine-tuning on our data. The NASR-baseline corresponds to the [42] case described in (23), while NASR-hybrid and NASR-higher-order respectively stands for using NASR augmented input with our Hybrid and Higher-order solving approaches.

***Joint inference with NASR***

Table 6 outlines the performance of different solving approaches, enhanced with NASR. The grid accuracy after reasoning for the standard NASR approach, albeit low in runtime, suffers from solely reasoning on most likely values. This solving approach is required during training to calculate a usable reward, as described in [42]. However, at test time, this causes the framework to fail finding any solution for $78.71\%$ of instances, on average. Switching to probabilistic constraint solving not only reduces the infeasibility rate down to $0\%$, but also improves the rate of correctly solved instances by $40\%$ on the Sudoku Assistant Data. Note that, although the Higher-order solving approach still improves accuracy over the baseline, it fails to do so with regard to the Hybrid approach. This under-performance can be explained by the way in which the repeated solving is triggered, for higher-order solving. As described in (), in the case of visual Sudoku, the uniqueness property requires perception variables to lead to a unique solution. The construction of $\tilde{Z}$ prevents any perception variables of getting assigned the blank value within the time limit.

### RQ4: detection and correction of user mistakes

We now focus on performing joint inference with constraint violation in the input, arising from human mistakes. In this section, we exclusively consider Sudoku Assistant data, as it contains both printed and handwritten values, and rely on a *Cell-CNN* that can classify both digit value and font style. We will compare the potential benefit of different ways of enriching our constraint solving model with additional properties about each digit, then evaluate the hybrid solver’s ability of correcting erroneous input from a human user.

**Fig. 8:**
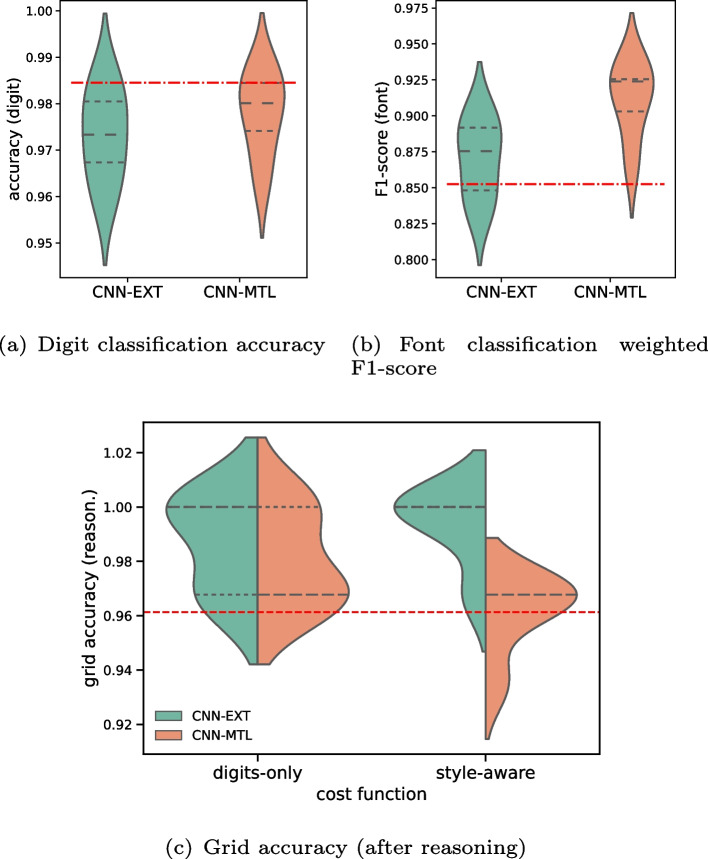
Violin plots of *Cell-CNN* architectures for joint digit classification and font classification on Sudoku assistant data, over 5 seeds. Dashed black line within violins divide each distribution into quartiles. Dashed red line indicates the mean performance of training a single *Cell-CNN* for each task separately

#### Font style classification

Erroneous visual input at inference time may lead to an unsatisfiable problem in the Visual Sudoku case. Assuming that these errors are mostly due to user mistakes, the machine learning classifier should learn to distinguish human input from initial givens, with the former being more error-prone. To that end, we consider two variants of the *Cell-CNN* architecture, introduced in Section 4.1.1. CNN-EXT is trained with an extended number of classes to account for printed and handwritten values, while CNN-MTL consists of backbone shared by two distinct multi-layer feed-forward networks. As done in standard multi-task learning settings, heads of the resulting *Cell-CNN* are trained in parallel. One head focuses on digit classification and the other addresses font style classification.

***Experimental setup***

Both architectures were trained for a maximum of 100 epochs, with a pre-trained VGG16 backbone and early stopping. We used the AdamW [67] optimizer, with a weight decay of 0.01 and a learning rate of $1\times 10^{-3}$. Figure 8 shows the results of the comparison. Both architectures are calibrated using: matrix scaling for Cell-Ext and temperature scaling for each head of the cell-mtl architecture. In the figure, *digit accuracy* refers to the proportion of cells whose numerical value is properly classified by the *Cell-CNN*. We use *weighted* F1-score as a performance indicator for font classification, to account for imbalance in the data with respect to printed and hand-written digits. As a baseline, we consider single *Cell-CNN*s trained on each task separately. Note that for digit classification, this baseline is equivalent to the setting in Section 7.3.

***CNN architecture***

For the digit classification task, both considered architectures fail to beat the baseline and they achieve comparable accuracy, as shown on Fig. 8(a). In contrast, they outperform the baseline on font classification, with Cell-MTL leading the way with an average weighted F1-score of $90.23\%$. Regardless of the architecture, the inclusion of the font classification task appears to train the CNN in such a way that their prediction lead to solutions of better quality, as illustrated by the left side of Fig. 8(c).

***Cost function***

Figure 8(c) also highlights the benefit of taking the additional font-style information into account when solving visual Sudokus, as shown by a lower variance and higher grid accuracy after reasoning, when considering a style-aware cost function.

**Fig. 9:**
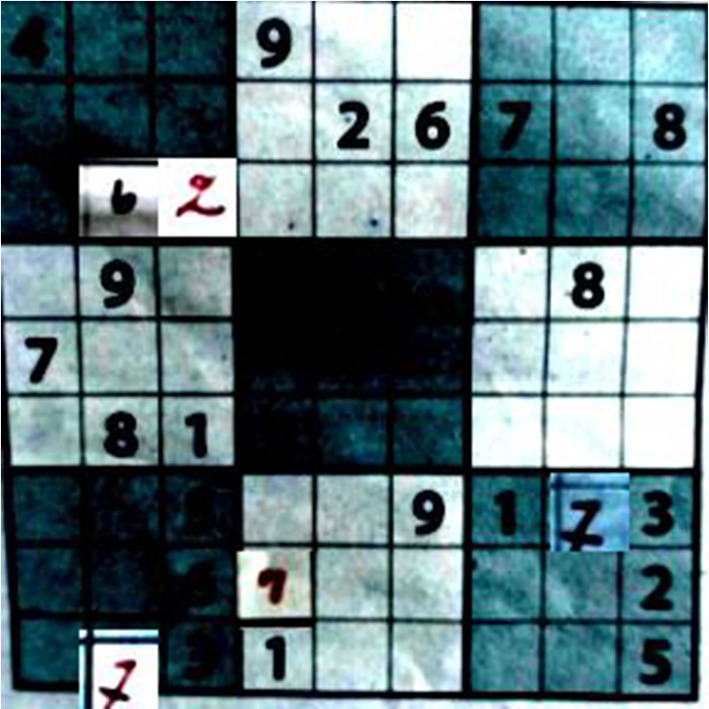
Example of an altered test sample, containing 5 erroneous handwritten digits sampled from the test set

#### Integrating font style into the CP model for correction

We now study the impact of input containing constraint violations on our framework capability to find the correct solution. In Section 6, we proposed two distinct approaches to address this challenge, assuming that erroneous sudokus are the consequence of user errors. The style-aware cost function approach consists of including a term in the cost function of the solver, accounting for handwritten digits during the search. The style-aware prior approach assumes a prior probability of user error, which depends on the likelihood for each digit to be handwritten. The solver is enhanced with a new set of constraints, enabling the assignment of a wildcard option, for cells whose probability scores do not surpass the prior. We empirically compare the solution quality of both approaches, against a baseline consisting of solving the puzzle without any additional font style information.

***Experimental setup***

Because *Cell-CNN*s provide cell-level predictions, we can easily generate faulty visual puzzles from valid instances by sampling handwritten images from the test set, and blending them within the original image at a wrong location (see Fig. 9). For each approach, we evaluate the grid accuracy after reasoning with $n \in \{0,1,2,5\}$ errors using the higher-order solving method.

***Performance analysis at low error rate***

**Fig. 10:**
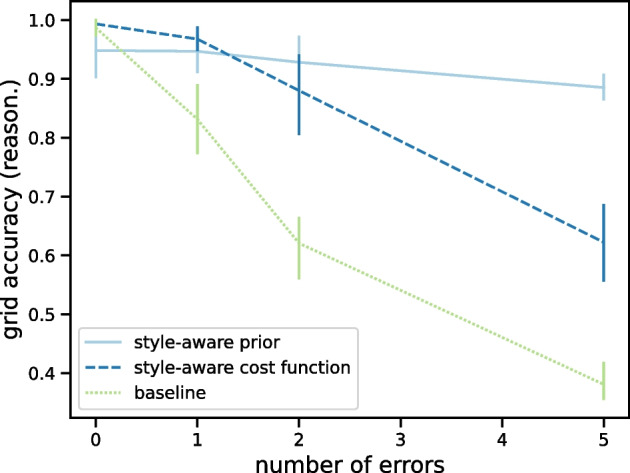
Comparison of methods for handling user mistakes, with varying number of erroneous input

We already observed the benefit of using style-aware cost function in the absence of errors, in Section 7.7.1. The style-aware prior approach initially leads to a drop in accuracy, but as we get to using it on faulty instances, its performance remains consistent, while the baseline accuracy drops by more than 10% (Fig. 10).

***Performance analysis at higher error rate***

The baseline performance is severely hindered as the number of errors increases, indicating the solver’s inability to correct errors. The style-aware cost function method (19) alleviates this by allowing the search to focus on printed digits. They are not prone to user errors as they usually form the initial givens of the puzzle. However, this strategy depends on the CNN to correctly classify font styles, which is not perfect, as shown in Fig. 8(b). Thanks to the wildcard option, the style-aware prior strategy delegates the choice of discarding cell probabilities to the solver. This approach, combined with higher-order reasoning, allows the solver to recover from user inputs in a more robust manner, maintaining $~90\%$ accuracy when up to 5 errors are introduced. This method also has the added benefit of not requiring any change to the cost function.

## Conclusion

Working with real-life Sudoku pictures poses several practical challenges, which we address in this paper. First, cell-level predictions are harder due to the inherent noise of real-life images. Furthermore, we assume a more challenging setting where the locations of the givens are not known. Finally, we also consider images of Sudokus partially filled by humans, meaning they might contain mistakes.

In our framework, the perception module is a shared CNN trained at the cell level, capable of classifying digit value and font style for each cell. In the reasoning module, we introduce the notion of perception variables and connect them to the decision variables of the Sudoku problem, with channeling constraints. The cost function maximizes the log likelihood of the CNN predictions to determine the maximum a posteriori assignment of decision variables. Furthermore, we consider the possibility of a mistake by the user while solving, leading to a partially filled Visual Sudoku that violates constraints due to the mistake. We address it by handling printed and hand-written digits separately, namely by assigning them different weights in the cost function, or by providing an option to the solver to ignore handwritten cells and infer their value through reasoning instead.

Our experiments show that, for Visual Sudokus, considering CNNs that operate at the cell level with shared weights instead of the whole image is beneficial. Indeed, it allows for a lighter trainable architecture combined with a pre-trained backbone and leads to an increasing number of solved instances. Our experiments on the OpenSky and MNIST datasets show the positive effect of calibration on solution quality. Our experiments on data from the Sudoku Assistant app demonstrated that a cost function that relies on the output of a font classifier to weigh human input and puzzle differently allows for a more robust model that can handle multiple user mistakes.

***Key takeaways***

The framework presented in this work contains several elements that are transferable to other applications combining perception and reasoning. One key transferable component is the interface between machine learning and constraint programming, implemented through perception variables, channeling constraints, and predictions encoded in the cost function. In PBCS problems with complete input, perception variables are the only variables required for reasoning. This is not the case in problems with partial input, where they interact with decision variables. Thus, they can differ in their domain, as shown here with Visual Sudoku solving, but also in their number. As an example, consider the task of reasoning over detected objects. Declaring one perception variable per detected object would work if there are no false negatives. Similarly to our addition of a *wildcard* value, and the modification of the channeling constraints introduced in (21), we could define a more robust CP modeling, to infer those missing detections. Namely, altering channeling constraints and the perception domains such that we would declare more perception variables than detected objects would allow the solver to recover from such misdetections. This exemplifies the applicability of the idea of modifying the channeling constraints to account for some potentially erroneous input, as introduced in Section 6.2.2, to other tasks.

The recognition of printed or hand-written digits is specific to perception-based constraint solving of pen-and-paper puzzles. However, the underlying idea of using multiple sensed properties to weigh the perception variables in the cost function differently is applicable to other problems. This idea can be applied to settings where reasoning on these additional properties can improve the accuracy of the system. As an example, a neural-based chessboard recognition system [68] enriched with constraint solving can benefit from incorporating shapes or color properties in the cost function, when identifying black or white pieces, respectively.

The solving strategy of reasoning over the top-k most likely values from the domain of perception variables is particularly important for time-sensitive applications, such as rendering the solution to a user with minimal delay [19, 69], or any application that requires repeated solving such as embedding a constraint solver in the training loop of a neural network, for constraint-aware learning [36, 42].

***Future work***

While the present work is set in the context of solving Sudokus from their images, it can be extended to other applications, where perception and reasoning can be combined to produce outputs that should satisfy certain known constraints. Examples include reconstructing scanned documents, estimating crop yields from satellite images [70, 71], visual question answering with known structures, scene graph parsing [72, 73], and many more.

We assumed a supervised learning setting, with full information from perception labels, which requires a non-negligible labeling effort. We could leverage knowledge about the reasoning task to generate labels in a weakly-supervised setting [74, 75]. Besides, this work integrates learning and reasoning only during the inference stage. Interesting avenues for future work include integrating learning and reasoning during the training of the neural network, i.e., training the neural network and the CSP end-to-end [36, 76]. In such a setting, joint inference becomes a bottleneck for learning. There has been recent success in using logic circuits for tractable inference in learning and reasoning tasks [77]. These circuits encode constraints as decision diagrams through knowledge compilation methods. While this allows for fast neuro-symbolic inference, the cost of constructing these diagrams increases as the constraints of the reasoning task become more complex. At test time, our *top-k* heuristic has shown to speed up the time needed to solve the problem, which could make such approaches more feasible on this problem.

## Data Availability

The datasets used in the experiments is publicly available[64], and the code to reproduce the experiments is available at https://github.com/CryoCardiogram/perception-based-constraint-solving.

## A Perception-based constraint solving with LLMs

Large Language Models (LLMs) are becoming increasingly better at solving reasoning tasks from a description in natural language, particularly with the advent of advanced prompting techniques such as Chain of Thought [78], In-context Learning [79]. More recently, Multi-modal LLM agents, equipped with vision encoders, can parse and reason about visual inputs [80]. As such, they are a prime candidate for Visual Sudoku Solving.

Let us consider as input a partially filled pen-and-paper sudoku board, similar to instances used in this paper. At the perception stage, multi-modal LLMs appear to accurately interpret the image, but can still make mistakes on more ambiguous symbols.

At the reasoning stage, LLMs attempt to fill in the grid according to the rules and can produce a complete solution. However, closer inspection reveals that this final solution is often invalid. This can be mitigated by asking the agent to proceed sequentially and to self-reflect on its solution at each step, but the observation remains. Besides, even in the case of a valid solution generated, the LLM can hallucinate during puzzle solving, and produce a solution, which starting clues differ from those of the input puzzle.

The LLM generates a solution by inferring the next most likely token, based on the internal representation of its training data. It is not equipped with any proper reasoning engine. Recently, ToolFormer [81] introduced the promising idea of training LLMs to delegate sub-tasks to external APIs. At the vision, this allows a multi-modal LLM agent to more accurately interpret the puzzle by generating a script that accurately partitions the input image into 9x9 patches, before processing them with a dedicated OCR library.

A similar approach could enhance the LLM reasoning capability, by delegating the sudoku solving to a specialized solver. This task is challenging, as it requires the LLM to provide information in a format suitable for the solver [82].

## B Perception

### B.1 Whole-CNN

We take inspirations from NeurASP’s [34] ’Sudokunet’ to build our Whole-CNN. The input layer consists of 5 successive convolutional block, each followed by batch normalisation, ReLU activation and Dropout. This layer embed the input image into a latent vector of size 512. The output layer is a global average pooling layer [83]. This layer performs a pooling operation that replace fully connected layers in classical CNNs. Global average pooling allows us to train the CNN on input images of any size.

### B.2 Cell-CNN

This section describes pre-trained architecture used as backbone for our Cell-CNN. When using a pre-trained backbone, we replace their output layer by a trainable 2-layer fully connected network, with 512 hidden nodes, Batch Normalisation, ReLU activation and Dropout.

***LeNet***

This CNN [37], pre-trained on MNIST [18], expects an image of size $28 \times 28$ as input. As handwritten digits in MNIST are in black-and-white, we apply adaptive thresholding [84] to binarize input images if required.

***VGG16***

Denotes the first five convolutional layers of the eponymous CNN [85], pre-trained on the Street View House Number [46]. Texpects input of size $32 \times 32$.

***ResNet18-32***

Resnets [6] include residual connections allowing them to be deeper while having fewer trainable parameters. They were pre-trained on ImageNet1K [86], with input images of size $224 \times 224$.

These networks are often used as backbones and clearly differ in the input dimensions, the color profile and the types of images they were trained on. We always use the normalization values provided with the dataset on which it was pre-trained; normalization is the process of shifting the input values to be zero-centered and scaled between $[-1, 1]$, which is known to improve convergence [87]

## C Experimental setup

***Hardware***

All neural networks were trained on a computer with a processor AMD EPYC 7502 32-Core Processor, and a GPU NVIDIA RTX A5000. Evaluations at test time were performed on a computer with a CPU Intel Core i7-8565U running at 1.80GHz, without GPU.

***Hyperparamters***

To fine-tune our constraint solving approaches, we relied on Bayesian optimisation, through its implementation in SMAC3 [88].

The style-aware cost function method (19) is parameterized by the coefficient $\alpha \in [0,1]$. The style-aware prior method (21) is parameterized by the coefficient $\gamma _e \in [0,1]$. We fine-tuned $\alpha$ and $\gamma _e$ on the validation set, by generating instances containing 1 error. We selected $\alpha =0.614$, and $\gamma _e = 0.793$.

## Footnotes

1 http://www.opensky.ca/~jdhildeb/software/sudokugen/
